# Excitation-inhibition imbalance disrupts visual familiarity in amyloid and non-pathology conditions

**DOI:** 10.1016/j.celrep.2022.111946

**Published:** 2023-01-04

**Authors:** Suraj Niraula, Julia J. Doderer, Shreya Indulkar, Kalen P. Berry, William L. Hauser, Oliver J. L’Esperance, Jasmine Z. Deng, Griffin Keeter, Adam G. Rouse, Jaichandar Subramanian

**Affiliations:** 1 Department of Pharmacology and Toxicology, School of Pharmacy, University of Kansas, Lawrence, KS 66045, USA; 2 Division of Experimental Hematology and Cancer Biology, Brain Tumor Center, Cincinnati Children’s Hospital Medical Center, Cincinnati, OH, USA; 3 Department of Neurosurgery, University of Kansas Medical Center, Kansas City, KS 66103, USA; 4 Present address: Department of Molecular Pharmacology and Experimental Therapeutics, Mayo Clinic, Rochester, MN 5590, USA; 5 Present address: Bioengineering Graduate Program, University of Kansas, Lawrence, KS 66045, USA; 6 Lead contact

## Abstract

Neuronal hyperactivity induces memory deficits in Alzheimer’s disease. However, how hyperactivity disrupts memory is unclear. Using *in vivo* synaptic imaging in the mouse visual cortex, we show that structural excitatory-inhibitory synapse imbalance in the apical dendrites favors hyperactivity in early amyloidosis. Consistent with this, natural images elicit neuronal hyperactivity in these mice. Compensatory changes that maintain activity homeostasis disrupt functional connectivity and increase population sparseness such that a small fraction of neurons dominates population activity. These properties reduce the selectivity of neural response to natural images and render visual recognition memory vulnerable to interference. Deprivation of non-specific visual experiences improves the neural representation and behavioral expression of visual familiarity. In contrast, in non-pathological conditions, deprivation of non-specific visual experiences induces disinhibition, increases excitability, and disrupts visual familiarity. We show that disrupted familiarity occurs when the fraction of high-responsive neurons and the persistence of neural representation of a memory-associated stimulus are not constrained.

## In brief

Niraula et al. show that neuronal hyperactivity in amyloidosis disrupts network architecture and reduces stimulus selectivity. Consequently, non-specific visual experiences interfere with visual recognition memory. In contrast, under non-pathological conditions, non-specific visual experiences constrain hyperactivity and improve visual recognition memory.

## Graphical Abstract

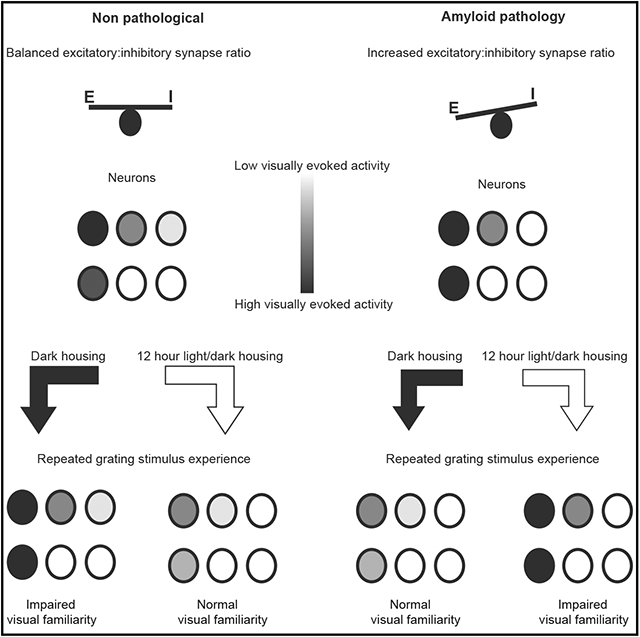

## INTRODUCTION

Structural synapse loss, which would reduce neuronal activity, correlates strongly with cognitive decline.^1,2^ However, neuronal hyperactivity is observed in the early stages of Alzheimer’s disease (AD) and mouse models of amyloidosis, suggesting excitation-inhibition (E/I) imbalance.^3-5^ Neuronal hyperactivity may occur, despite structural synapse loss, due to increased glutamatergic, reduced GABAergic transmission, or elevated intracellular calcium release.^6-20^ Alternatively, structural changes, such as an imbalance of excitatory and inhibitory synapse densities,^21-23^ may promote hyperactivity in the early stages of amyloidosis. *In vivo* imaging studies of synapses in mouse AD models are restricted to dendritic spine imaging.^24^ Because not all dendritic spines carry mature excitatory synapses, and inhibitory synapses are missed during spine imaging, whether structural synaptic changes favor or oppose hyperactivity in early amyloidosis is unclear.

E/I imbalance-driven neuronal hyperactivity disrupts activity homeostasis and imposes energy constraints on cellular and circuit functions.^25,26^ Whether neurons retain the ability to achieve activity homeostasis in amyloidosis and how circuits are shaped by hyperactivity remain unclear. Neuronal hyperactivity-induced memory deficits may result from circuit adaptation^27^ or could stem from disruption of plasticity.^28-30^ However, how E/I balance regulates memory in non-pathological conditions and the mechanisms by which hyperactivity disrupts plasticity and memory in amyloidosis are poorly understood.

We address these knowledge gaps using *in vivo* imaging of synaptic architecture and microcircuit functional connectivity in the mouse visual cortex to uncover the cellular and network mechanisms of visual recognition memory in amyloidosis and non-pathological conditions. Visual recognition memory is impaired in patients with AD.^31,32^ In mice, visual recognition memory is measured by assessing their innate curiosity to explore novel but not familiar objects or visual stimuli. Mice show behavioral habituation selectively to a repeatedly experienced grating stimulus of specific orientation.^33,34^ Orientation-specific recognition memory is dependent on plasticity in the visual cortex.^35^ Although the visual cortex does not display overt neurodegeneration early in AD, it is vulnerable to amyloid accumulation in patients and AD models.^36-40^

Here we analyzed ~20,000 synaptic structures in living animal brains and show that the excitatory-inhibitory synapse ratio in dendrites increases in amyloidosis without altering average synaptic density. The ensuing hyperexcitability alters network architecture, interferes with formation of efficient neural correlates of familiarity, and disrupts the behavioral expression of visual recognition memory. Deprivation of non-specific visual experiences improved memory in amyloidosis but disrupted it in non-pathological conditions despite normal plasticity (based on multiple metrics). In non-pathological conditions, deprivation of non-specific visual experiences induced disinhibition and increased the persistence of neural representation and the fraction of high-responsive neurons to the repeatedly experienced stimulus, leading to impaired memory. These results indicate that hyperactivity induces recognition memory deficits, despite normal synapse density and plasticity, by preventing formation of the neural representation of familiarity.

## RESULTS

### Localized structural E/I imbalance in dendrites of layer 2/3 neurons in the visual cortex

**Figure 1.**
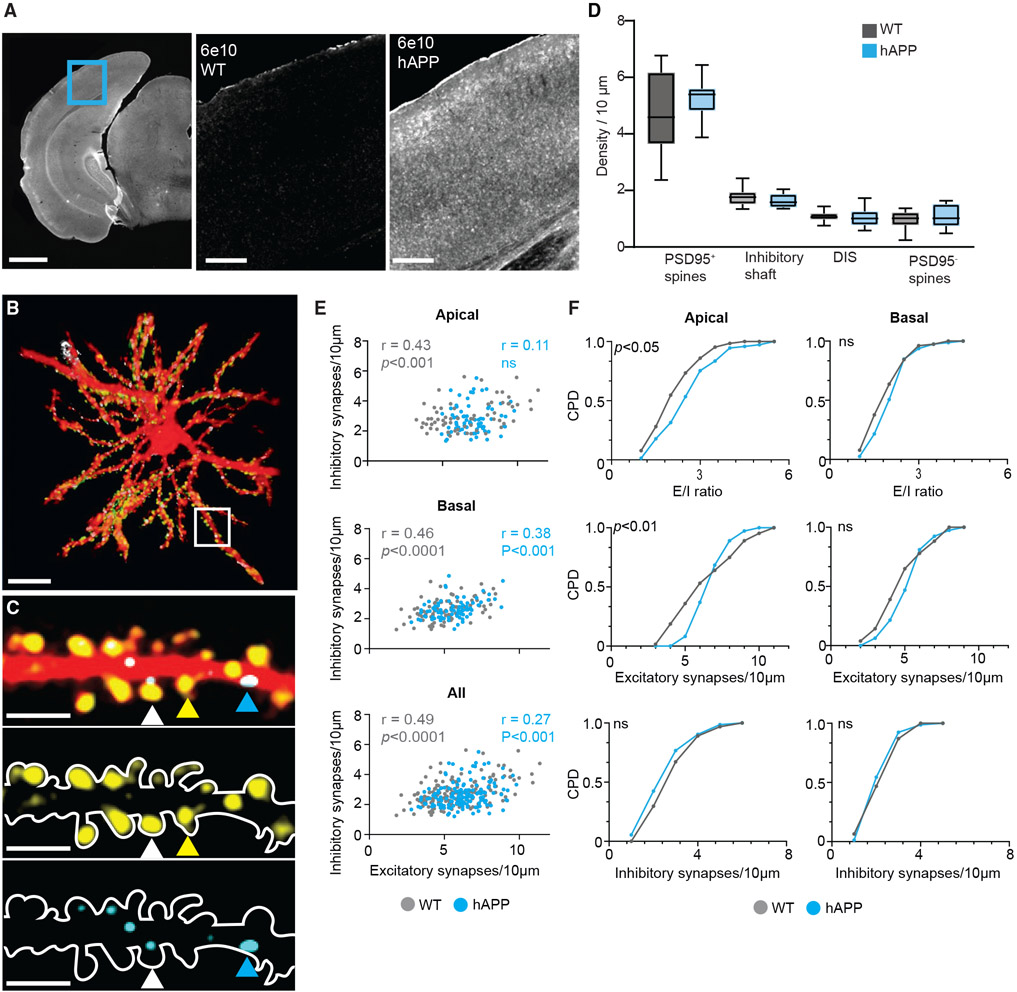
Structural E/I imbalance in amyloid pathology (A and B) A representative image of a posterior cortical slice (A, left; scale bar, 1 mm). The blue box represents part of the visual cortex. Shown is amyloid immunohistochemistry with the 6e10 antibody in wild-type (WT; center) and hAPP (right) slices (identical display range). Scale bars, 200 μm. Also shown (B) is a top-down view of a 3D reconstructed layer 2/3 neuron *in vivo*. Scale bar, 20 μm. A mask was applied to isolate the neuron from autofluorescent structures in the brain. The dendritic segment in the white square is magnified in (C). (C) Pseudocolored dendritic segment containing dendritic spines with PSD95 (yellow arrowhead), PSD95, gephyrin (white arrowhead), and shaft gephyrin (blue arrowhead). PSD95 (center) and gephyrin (bottom) channels are shown. Scale bars, 5 μm. (D) The density of synaptic structures: spines with PSD95 (PSD95^+^), inhibitory synapse on the shaft, dually innervated spines (DISs) with PSD95 and gephyrin, and spines without PSD95 (PSD95^−^). Data are presented as box (25th—75th percentile) and whisker (minimum and maximum values) plots, and median value is indicated as a horizontal line. n = 11 cells from 10 mice (WT) and 12 cells from 12 mice (hAPP). (E) Pearson correlation (r) between excitatory and inhibitory synapse density for apical (n = 64 [WT, p < 0.001] and 73 [hAPP, p > 0.05] dendrites, top), basal (n = 77 [WT, p < 0.0001] and 79 [hAPP, p < 0.001] dendrites, center), and all (n = 141 [WT, p < 0.0001] and 152 [hAPP, p < 0.001] dendrites, bottom) dendrites. (F) Cumulative probability distribution (CPD) of the same dendritic segments (left, apical; right, basal) for E/I ratio (top), excitatory synapse density (center), and inhibitory synapse density (bottom). p < 0.05 (E/I ratio, apical), p < 0.01 (excitatory synapses, apical), Kolmogorov-Smirnov test; ns, not statistically significant. See also Figures S1-S3.

We found that mice overexpressing human amyloid precursor protein with mutations linked to familial AD (hAPP mice; J20 line^41^) express high levels of amyloid but not plaques in the visual cortex at 4–6 months of age used for our experiments (Figure 1A). Also, we found increased c-Fos immunolabeling in the visual cortex of hAPP mice, indicative of hyperactivity (Figure S1A). c-Fos immunofluorescence did not significantly correlate with amyloid immunofluorescence detected by the 6e10 antibody (Figures S1B-S1F).

To identify whether structural synaptic changes favor (increased excitatory or decreased inhibitory synapses) or oppose (decreased excitatory or increased inhibitory synapses) hyperactivity, we used a synaptic labeling strategy that reliably represents excitatory and inhibitory synapses *in vivo*.^42-44^ We achieved sparse labeling of individual cortical neurons by combining low levels of a Cre recombinase (Cre)-expressing plasmid alongside high levels of Cre-dependent plasmids in a lentiviral backbone that expresses (1) TdTomato to visualize spines, (2) postsynaptic density-95 (PSD-95) tagged with Venus fluorescent protein at the C terminus (PSD95-Venus) to visualize mature excitatory synapses, and (3) gephyrin tagged at the N terminus with teal fluorescent protein (teal-gephyrin) to visualize inhibitory synapses (Figures 1B, 1C, and S2A-S2D).

We analyzed ~20,000 synaptic structures (~23,000 μm of total dendritic length) from apical and basal dendrites of layer 2/3 neurons in wild-type (WT) and hAPP mice *in vivo*. Excitatory and inhibitory synapse densities in non-transgenic controls (WT) are comparable with those observed previously^43,44^ (Figure 1D). Furthermore, the average synapse densities of neurons in hAPP mice did not differ from the WT in the visual cortex at around 6 months of age (Figure 1D). Averaging synapse densities from multiple dendrites will not reveal the balance of excitatory and inhibitory synapses on individual dendrites. Simultaneous imaging of excitatory and inhibitory synapses allowed us to determine their relative densities in the same dendrites. We found that the densities of excitatory and inhibitory synapses in WT mice are significantly correlated in apical and basal dendrites, indicating structural E/I balance (Figure 1E). However, the E/I correlation is not observed in apical dendrites of hAPP mice (Figure 1E). Furthermore, the correlation in apical dendrites of WT (r = 0.43) and hAPP (r = 0.11) mice is significantly different (p = 0.02, Fisher r-to-z transformation). In contrast, basal dendrites of hAPP mice retained a significant E/I correlation (Figure 1E). E/I imbalance in apical dendrites of hAPP mice results from a higher E/I ratio in these dendrites compared with WT mice (Figure 1F). The E/I ratio of basal dendrites did not differ between the genotypes. The overall changes to the density of excitatory and inhibitory synapses (5 more and 1 fewer synapses per 100 μm in hAPP mice, respectively) are subtle.

To test whether exogenous expression of synaptic markers prevented the loss of synapses in hAPP mice, we performed immunohistochemistry and compared PSD95 and gephyrin puncta in the visual cortex of WT and hAPP mice (Figures S3A-S3C). We observed a small but not significant increase in PSD95 puncta and no change in gephyrin puncta in hAPP mice compared with the WT (Figures S3B and S3C), consistent with our imaging data and other studies that show no change or increased excitatory synapse densities in early stages of amyloidosis.^45-47^

To address whether apical and basal dendrite differences in the E/I ratio occur in other brain regions, we compared PSD95 and gephyrin puncta in the hippocampal CA1 region, which displays dendritic hyperactivity^15,48^ in amyloidosis. CA1 has anatomically distinguishable apical (*stratum radiatum*) and basal (*stratum oriens*) dendritic domains. In the *stratum radiatum*, PSD95 and gephyrin punctum densities were not significantly different, but their ratio was increased significantly compared with WT mice (Figures S3D and S3E). In contrast, in the *stratum oriens,* we found that PSD95 puncta were significantly higher but not gephyrin puncta, leading to an increased PSD95-gephyrin ratio (Figures S3F and S3G). These results show that the increased E/I ratio is not restricted to apical dendrites. Together, our findings identify that structural synaptic changes favor hyperactivity in early amyloidosis.

### Stimulus-selective hyperactivity in the visual cortex of hAPP mice

**Figure 2.**
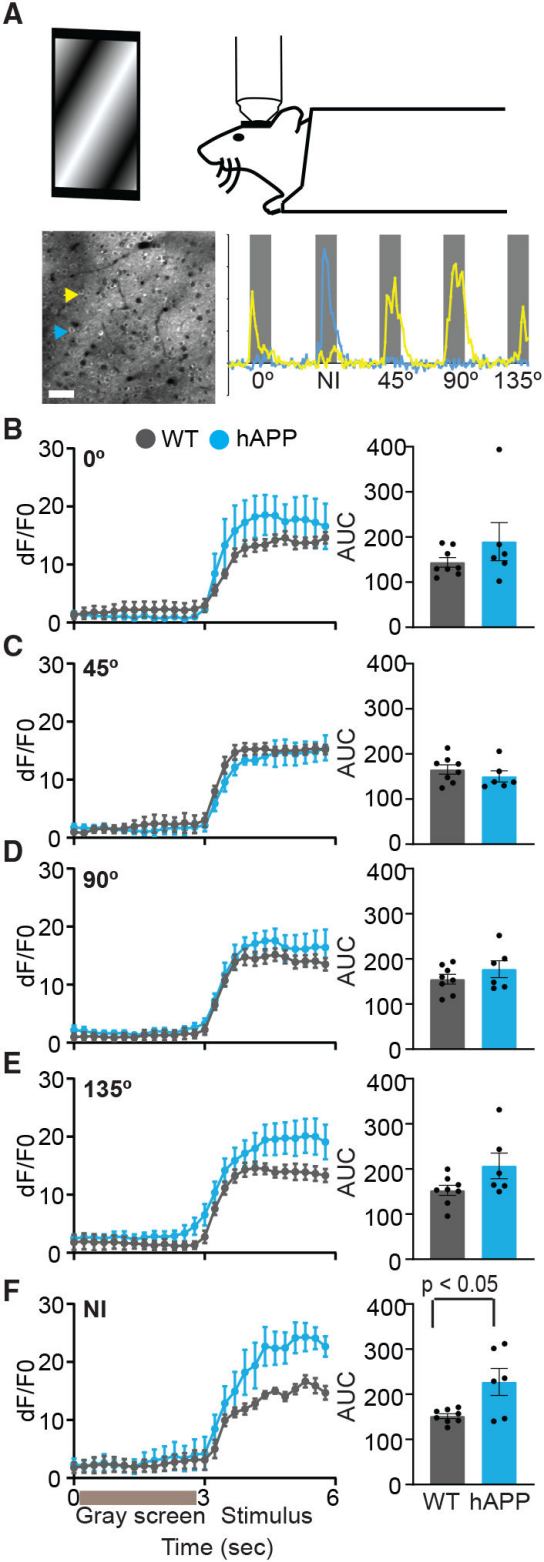
Natural images elicit hyperactivity in hAPP mice (A) Illustration of a head-fixed mouse viewing a visual stimulus (top). Representative imaging field (bottom left; scale bar, 50 μm) and calcium transients of neurons (yellow and blue arrowheads) during the first cycle of stimulus (bottom right). Gray bars, stimulus duration; NI, natural image. (B–F) Average dF/F0 during 3-s gray screen and stimulus periods (left) and the area under the curve (AUC) for the 3-s stimulus period (right) for neurons considered active for 0° (B), 45° (C), 90° (D), 135° (E), and NI (p < 0.05, unpaired Student’s t test; F) stimuli in each animal. Data are mean ± SEM. n = 8 (WT: 91 [0°], 99 [45°], 141 [90°], 95 [135°], and 151 NI neurons) and 6 (hAPP: 60 [0°], 46 [45°], 58 [90°], 52 [135°], and 71 NI neurons) mice. Circles in the histogram represent individual mouse values. See also Figures S4 and S5.

To study the functional consequences of hyperactivity, we crossed hAPP mice with transgenic mice expressing fluorescent calcium indicator GCaMP6 and measured visually evoked calcium transients. We presented four phase-reversing orientation gratings (0°, 45°, 90°, and 135°) and two sets of 10 natural images in a random order, separated by gray screen, to awake head-fixed mice (Figure 2A). We classified neurons as active to the stimulus when the trial-averaged (8 cycles) dF/F0 (change in fluorescence over the baseline fluorescence) during the stimulus period exceeded a threshold (Figures 2B-2F, high threshold; Figure S4, low threshold; STAR Methods). The area under the curve (AUC) of trial-averaged dF/F0 for neurons classified as active was moderately higher for certain orientations (0° and 135°) in hAPP mice compared with WT mice but reached statistical significance only with the low-threshold criterion (Figures 2B-2E and S4D). In contrast, natural images elicited a significantly higher response in neurons classified as active in hAPP mice regardless of the threshold criteria (Figures 2F and S4D). These results show that natural images and some grating stimuli evoke hyperactivity in neurons. However, we noticed that the average number of neurons identified for analysis and the number of neurons responsive to visual stimuli was lower in hAPP mice (Figure S5A). Although the reduction was not significant with the high-threshold criterion, it reached statistical significance when the threshold for activity was lowered, indicating that weakly responsive neurons are reduced in hAPP mice (Figure S5A). Similarly, the fraction of all identified neurons considered active (high threshold) for each stimulus did not differ between the genotypes (Figure S5B), whereas they were reduced for orientation grating stimuli when the threshold for active neurons was lowered (Figure S5C).

**Figure 3.**
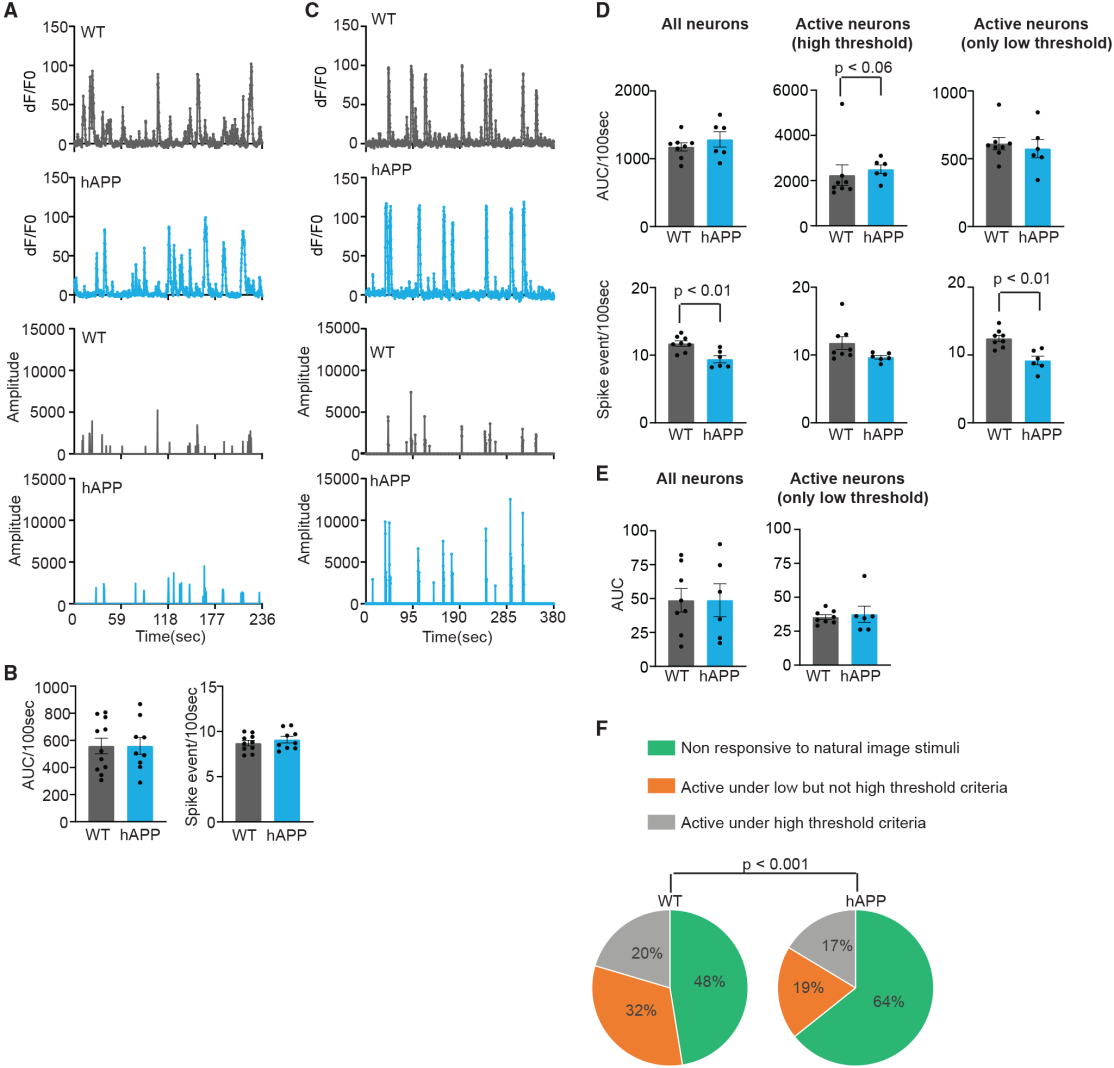
Reduced activity in low-responding neurons compensates for hyperactivity (A) Representative traces of calcium transients and corresponding deconvolved spike trace of spontaneous activity from WT and hAPP mice. (B) Average AUC and the number of deconvolved spikes (spike events) in every 100 s of spontaneous activity from all identified neurons for each animal. n = 10 (WT, 860 neurons) and 9 (hAPP, 780 neurons) mice. (C) Representative traces of calcium transients and corresponding deconvolved spike trace across the entire imaging session of evoked activity (multiple stimuli) from WT and hAPP mice. (D and E) Average AUC (top) and the number of deconvolved spikes (spike events, bottom) in every 100 s of evoked activity across multiple stimuli (D). Shown are (left) all identified (740 WT and 434 hAPP neurons), (center) active for any stimulus (high-threshold criteria; 353 WT and 168 hAPP neurons), and (right) weakly responsive for any stimulus (active only when the activity criteria threshold is lowered; 349 WT and 171 hAPP neurons) from n = 8 WT and 6 hAPP mice. p < 0.01 (unpaired Student’s t tests, left and right bottom). Also shown (E) are the average AUC of trial-averaged dF/F0 (3 s) elicited by NI stimuli of all identified neurons (WT = 740, hAPP = 434 neurons; left) and neurons classified as active only when the threshold is lowered (238 WT and 84 hAPP neurons; right) from 8 WT and 6 hAPP mice. Data are mean ± SEM. Circles in the histogram represent individual mouse values. (F) Proportion of neurons that are not (351 WT and 279 hAPP), weakly (238 WT and 84 hAPP), or highly (151 WT and 71 hAPP) responsive to NI stimuli from all mice. p < 0.001 (chi-square test).

To test whether spontaneous activity is increased in hAPP mice, we imaged neuronal activity without visual stimulus for 237 s. The average AUC of dF/F0 (100 s) and the number of imaging frames with deconvolved spikes (spike events) calculated from all identified neurons did not differ between the genotypes (Figures 3A and 3B). This is consistent with our findings that the gray screen (Figure S5D) and some orientation gratings (Figure 2) did not show a difference between hAPP and control mice.

### Homeostatic regulation of population activity leads to hypersparsification of neural code and functional connectivity in amyloidosis

Neuronal activity homeostasis is tightly regulated to avoid runaway excitation.^49^ To test whether hyperactivity in hAPP mice is compensated over a longer period, we first compared the average AUC of dF/F0/100 s over the entire imaging period (consisting of a gray screen, gratings, and natural image stimuli; Figure 3C) in WT and hAPP mice. The active (high-threshold) neurons in hAPP mice still retained higher activity when averaged over a 100-s period compared with the WT (but p < 0.06). However, the average AUC obtained from all identified and weakly responsive (considered active using the low but not high-threshold criteria) neurons were similar (Figure 3D). Interestingly, in these neurons, the total number of spike events was reduced in hAPP mice. Reduced spike event frequency and equal AUC indicate that each spike event in these mice elicits a higher amplitude response and that the compensatory nature of reduced spike event frequency and increased amplitude per spike event maintains the total activity level in the population.

We next restricted the analysis to imaging frames corresponding to natural image stimuli to test whether hyperactivity in neurons classified as active (high threshold) to natural images (Figure 2F) is compensated at the population level. The average AUC of trial-averaged dF/F0 of all identified neurons did not differ between the genotypes (Figure 3E), suggesting that hyperactivity of active (high-threshold) neurons does not affect the total population activity elicited by natural images. To test whether it is due to compensatory reduction in the average AUC of weakly responsive neurons, we compared the average AUC of trial-averaged dF/F0 from neurons considered active with low but not high-threshold criteria and found that they remained the same between the genotypes (Figure 3E). However, we found that the fraction of weakly responsive neurons decreased, and nonresponsive neurons increased, in hAPP mice (Figure 3F). Consequently, the increased contribution of active (high-threshold) neurons to total population AUC (61% [WT] and 73% [hAPP] of total population AUC) was compensated by the reduction in the contribution of weakly responsive neurons (25% [WT] and 12% [hAPP] of total population AUC). The contribution of nonresponsive neurons to total AUC remained the same between genotypes (14%–15%). These findings reveal that neurons whose activity is weakly modulated by natural images become hypoactive to compensate for the hyperactivity of high-responding neurons in hAPP mice.

**Figure 4.**
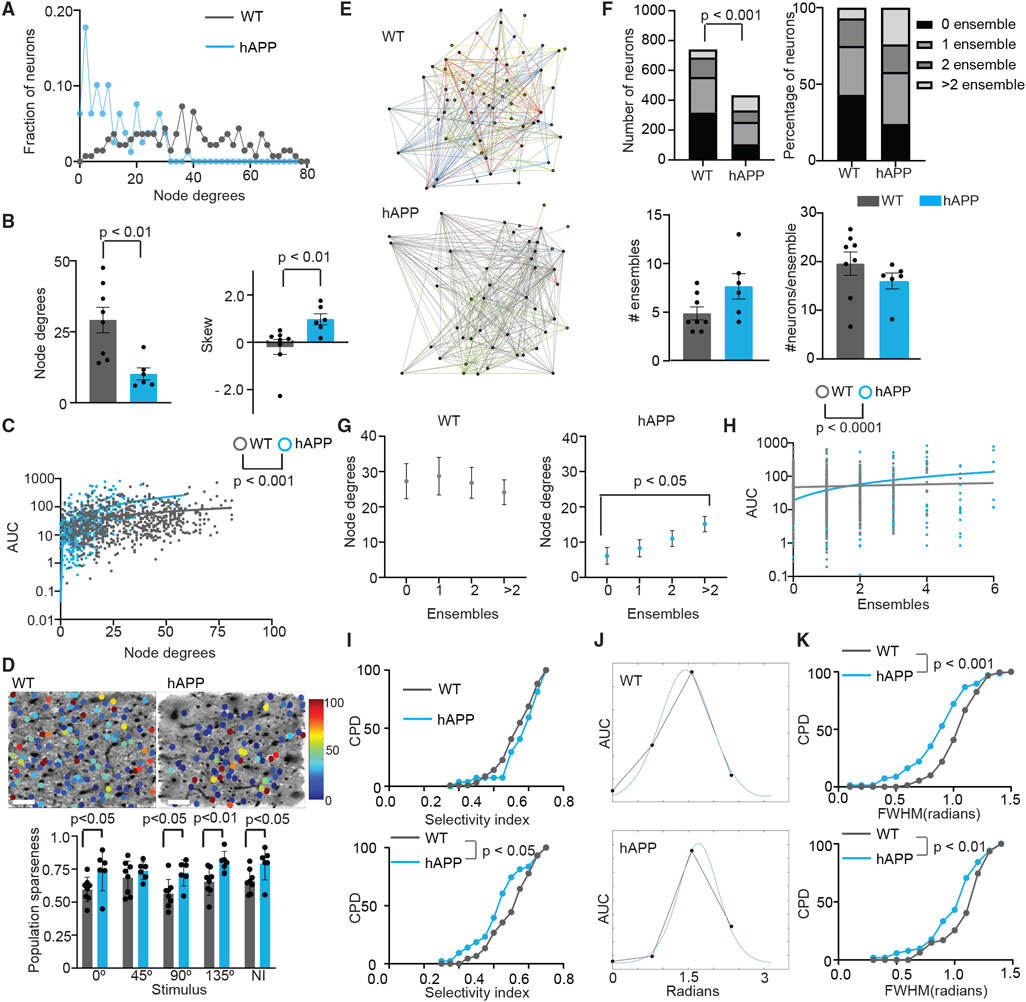
Altered functional connectivity and stimulus selectivity in hAPP mice (A) Representative degree distribution calculated during the entire imaging period across multiple stimuli from WT and hAPP mice. (B) Average node degree (left; p < 0.01, unpaired Student’s t test) and skew (right; p < 0.01, Mann-Whitney test) of distribution from all identified neurons from each animal. (C) Scatterplot comparing node degree and AUC during the 3 s of NI stimulus of all identified neurons (n = 740 WT and 434 hAPP neurons; y axis: log scale). Gray and blue lines (slopes, p < 0.001) are least-squares fits for WT and hAPP mice, respectively. Circles represent the values of individual neurons. (D) Top: representative images of activity distribution in WT (left) and hAPP (mice); scale bars, 50 μm. Filled circles represent neurons, and the color represents trial-averaged AUC (color bar, right) during the 3 s of NI stimulus. Bottom: population sparseness for grating and NI stimuli. p < 0.05 (0°, 90°, and NI) and p < 0.01 (135°), unpaired Student’s t tests. (E) Representative ensemble from WT (top) and hAPP (bottom) mice. Colors represent ensemble identity (gray, more than one ensemble). Circles represent cells. (F) Top left: number of all identified neurons in no, one, or multiple ensembles (n = 740 WT and 434 hAPP neurons, top left) across multiple stimuli. P < 0.001, chisquare test. Top right: the same data as the percentage of neurons. Also shown are the average number of ensembles (bottom left) and the average number of neurons per ensemble (bottom right) from each animal. (G) Relationship between the number of ensembles and node degrees for all identified neurons in WT (left) and hAPP (right; p < 0.05, Kruskal-Wallis test) mice. (H) Scatterplot comparing ensemble number and average AUC (y axis: log scale) during 3-s NI stimuli from all identified neurons. Gray and blue lines (slopes, p < 0.0001) are the least-squares fits for WT and hAPP mice, respectively. (I) CPD of NI selectivity index of neurons that are responsive to NI stimuli and participate in one or less ensemble (n = 128 WT and 27 hAPP neurons, top) or more than one ensemble (n = 45 WT and 43 hAPP neurons; p < 0.05, Kolmogorov-Smirnov [KS] test; bottom). (J) Representative examples of orientation tuning curves from WT (top) and hAPP (bottom) neurons. The black circles represent the average AUC during 3 s of 0° (0 radians), 45° (0.78 radians), 90° (1.57 radians), and 135° (2.35 radians) grating stimuli. The blue line represents the tuning curve. (K) CPD of full width at half-maximum (FWHM) of the tuning curve of neurons that respond to any grating stimuli and participate in one or less ensemble (n = 201 WT and 68 hAPP neurons; p < 0.001, KS test; top) or more than one ensemble (n = 47 WT and 51 hAPP neurons; p < 0.01, KS test; bottom). Data are mean ± SEM. n = 8 WT and 6 hAPP mice (B, D, and F, bottom). Circles in the histogram represent individual mouse values. See also Figures S6 and S7.

A reduced frequency of spike events (Figure 3D) may reduce the probability that two neurons are functionally connected or coactive in the same imaging frame (~250 ms duration). Neuron pairs are considered functionally connected when the number of imaging frames in which they are coactive is greater than 95% of the cumulative distribution of coactivation obtained from 1,000 random circular shifts of their activity. We calculated the node degree, which is the average number of significantly coactive neurons for each neuron, and compared their distribution in WT and hAPP mice during the entire imaging period consisting of a gray screen, gratings, and natural image stimuli. Neurons in WT mice show a higher node degree than hAPP mice, whose degree distribution is more positively skewed, indicating that very few neurons have high functional connectivity in hAPP mice (Figures 4A and 4B).

Skewed connectivity in hAPP mice could result from few hyperactive and many hypoactive neurons, with hyperactive neurons forming the most functional connections. Consistent with this, we found that the response to natural images increased with higher node degree, and the slope (significantly non-zero, p < 0.0001 WT and hAPP) of this increase was significantly higher for hAPP mice compared with WT mice (Figure 4C). We found similar results for orientation grating stimuli (Figure S6).

Higher activity of the few highly connected neurons could increase sparseness of the neural code. Sparse coding requires less energy to represent information.^50^ Therefore, we calculated population sparseness, a measure of the shape of neuronal activity distribution. The value ranges from 0–1, where 0 indicates equal activity among all neurons, and 1 indicates only one active neuron in the population. Consistent with previous findings,^51^ we found that population sparseness is high for most of the stimuli in WT mice (Figure 4D). Interestingly, it is even higher in hAPP mice (Figure 4D), indicating that neural activity is hypersparsified.

The skewed distribution of connectivity may alter the organization of neuronal subnetworks (ensembles; described in STAR Methods). To assess this, we assigned functionally connected neurons to distinct ensembles.^52^ Neurons can be part of zero, one, two, or more ensembles. Interestingly, a higher fraction of neurons in hAPP mice than in the WT participated in more than two ensembles. However, the total ensemble number and the number of ensembles were not different (Figures 4E and 4F). Because few neurons in hAPP mice are highly active and more functionally connected, these neurons may participate in multiple ensembles. Consistent with this, neurons that participated in more than two ensembles had more node degrees than those that participated in fewer ensembles in hAPP mice (Figure 4G), indicating that only a small fraction of neurons is highly relevant to circuit architecture. Furthermore, the AUC of response elicited by natural images is higher in neurons participating in multiple ensembles in hAPP mice (slope significantly non-zero, p < 0.0001) but stayed consistent in WT mice (Figure 4H). Our findings show that increased excitability of neurons, counterintuitively, leads to hypersparsification of neural activity and network connectivity and alters ensemble organization to maintain activity homeostasis.

We examined whether natural image-driven hyperactivity and altered circuit architecture reduce stimulus specificity. Because we delivered 10 different natural images in 3 s, we measured the similarity of responses to different natural images during the 3 s (13 imaging frames). We calculated a selectivity index (described in STAR Methods) ranging from 0 (all imaging frames had the same amplitude spike events [deconvolved], low selectivity) to close to 1 (only one of the 13 frames had a spike event [deconvolved], high selectivity). We found that neurons participating in multiple ensembles in hAPP mice showed lower selectivity of responses to natural images than controls (Figure 4I). In contrast, the full width at half-maximum of the orientation tuning curve decreased in hAPP mice, indicating higher selectivity for orientation grating stimuli. To test whether the same neurons show reduced selectivity for natural images and increased selectivity for orientation gratings, we repeated this analysis in neurons responsive to natural image stimuli and any of the grating stimuli. We found that these neurons also exhibit increased selectivity to orientation gratings and reduced selectivity to natural images (Figures S7A and S7B), and these two features are not correlated (Figure S7C). One mechanism that would sharpen orientation selectivity is biased connectivity between similarly tuned neurons.^53^ Consistent with this, functionally connected neurons in hAPP mice are more similarly tuned compared with controls (Figure S7D).

### Non-specific visual experiences differentially modulate the neural representation of familiarity in WT and hAPP mice

The altered functional responses in amyloidosis, such as reduced stimulus selectivity and skewed degree distribution, indicate that multiple natural visual experiences activate the same neurons. This could disrupt plasticity evoked by repeated experience of the same visual stimulus, leading to memory interference. Repeated exposure to a grating stimulus leads to orientation-selective plasticity in the visual cortex.^34,54-56^ Therefore, we measured GCaMP6s response to a 45° phase-reversing grating stimulus before and after multiple days of exposure (“training”) to the same 45° stimulus. Because plasticity is highly selective to orientation,^57^ we also tested plasticity to a 75° grating stimulus in the same mice, this time housed in complete darkness except during the stimulus period (Figure S8A). Under dark housing, mice only encountered specific visual experiences and were not susceptible to interference by non-specific visual experiences, which would be present during a 12-h light/dark cycle when the effect of 45° stimulus training was assessed.

**Figure 5.**
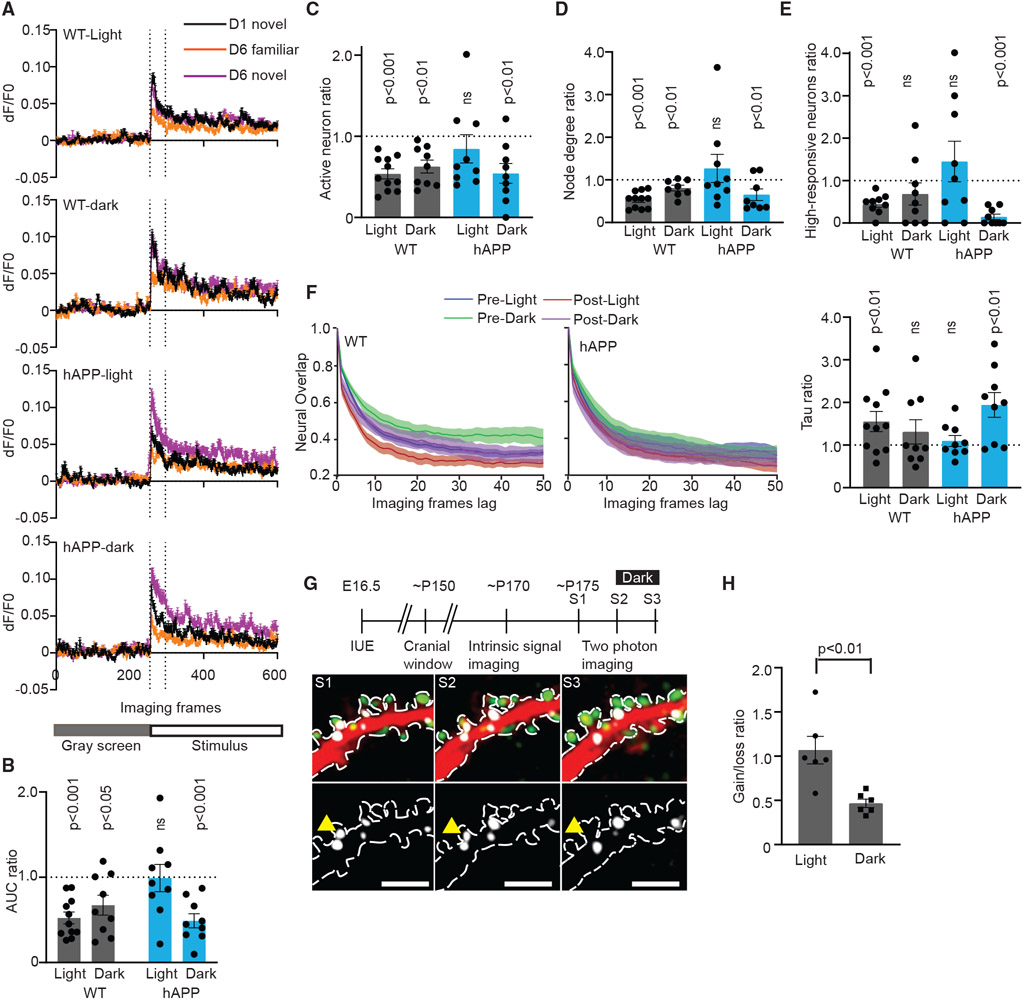
Nonspecific visual experiences differentially influence visual cortex plasticity (A) Average dF/F0 of calcium transients during the gray screen and stimulus. Dotted lines indicate the first 10 s of stimulus. (B) Average post- to pre-training ratio of AUC during the first 10 s of the same stimulus for all identified neurons from each animal (669 WT and 494 hAPP [pre-training-light], 584 WT and 538 hAPP [post-training-light], 469 WT and 518 hAPP [pre-training-dark], and 416 WT and 383 hAPP [post-training-dark] neurons). Values below the dotted line show reduction in AUC after training. (C) The average post- to pre-training ratio of the fraction of identified neurons classified as active during the first 10 s of stimulus. Values below the dotted line show reduction induced by training. (D) The average post- to pre-training ratio of node degrees (number of coactive neurons to each neuron) during the first 10 s of stimulus for active neurons. Values below the dotted line show reduction induced by training. (E) The average post- to pre-training ratio of the fraction of neurons with dF/F0 > 15% (high-responding neurons) during the first 10 s of stimulus. (F) The decay of neural overlap between imaging frames for up to 50 frames of separation for WT (left) and hAPP (center). Data were averaged for frames in the first 10 s of the stimulus. Also shown is the average post- to pre-training ratio of the rate of decay (Tau, right). Values above the dotted line show the increase in decay rate induced by training. WT-light: p < 0.001 (B–E), p < 0.01 (F); WT-dark: p < 0.05 (B), p < 0.01 (C and D), p > 0.05 (E and F); hAPP-light: p > 0.05 (B–F); hAPP-dark: p < 0.001 (B and E), p < 0.01 (C, D, and F), one sample t tests (comparison of the sample mean with the ratio of 1) or Wilcoxon signed-rank test (B–F). n = 11 (WT-light), 9 (WT-dark), and 9 (hAPP-light and dark) mice. ns, not significant (p > 0.05). For the data presented as ratios of post/pre-training, the pre- and post-training means are provided in Table S2. (G) Timeline for imaging gephyrin dynamics (top). IUE, *in utero* electroporation; E, embryonic; P, post-natal age. S1, S2, and S3 are imaging sessions separated by 1 week each. Dark bar, dark housing (bottom). Shown is a representative pseudocolored dendritic segment (merged, top; gephyrin, bottom) exhibiting loss of gephyrin (yellow arrowhead) between S2 and S3 (left). Scale bars, 5 μm. (H) Average gain/loss of gephyrin when mice were housed under a normal light cycle and 24-h darkness. p < 0.01, paired Student’s t test; n = 6 WT cells (1,000 synapses). Data are mean ± SEM. Circles in the histogram represent individual mouse values. See also Figure S8.

The post-training population (all identified neurons) response (AUC during the first 10 s of stimulus) is 55% and 67% of pre-training levels in WT mice under a 12-h light-dark cycle (WT-light) and 24-h dark housing (WT-dark), respectively, indicative of neuronal plasticity (Figures 5A and 5B). In contrast, hAPP mice did not show plasticity in light (hAPP-light; 99% of pre-training levels) but showed plasticity (49% of pre-training levels) in the dark (hAPP-dark; Figures 5A and 5B). In contrast to familiar stimuli, the response to novel stimuli did not reduce following training (Figure S8B). Similarly, the fraction of neurons classified as active during the first 10 s of stimulus exposure reduced after training in WT (light [post/pre ratio: 0.54] or dark [post/pre ratio: 0.62]) and hAPP-dark (post/pre ratio: 0.54) mice but not in hAPP-light mice (Figure 5C). These findings show that neuronal response plasticity in the visual cortex of hAPP mice is susceptible to interference from non-specific visual experiences.

The lack of reduction of active neurons in hAPP-light mice following training (Figure 5C) suggests that hyperactivity elicited by natural images in their home cage may prevent the weakening of functional connectivity of these neurons. Consistent with this, the average node degree of neurons for the trained stimulus reduced for hAPP-dark (post/pre ratio: 0.65) and WT light (post/pre ratio: 0.53) or dark (post/pre ratio: 0.80) mice after training but not for the hAPP-light group Figure 5D). In contrast, the node degree of neurons did not reduce under any conditions for the non-trained orientation for all groups (Figure S8C). A reduction in functional connectivity with repeated stimulus exposure may further increase population sparseness.^58^ Training further increased population sparseness in WT but not hAPP mice, whose circuit activity is already hypersparsified (Figure S8D).

We performed linear regression to test whether the reduction in population AUC ratio can be explained by the reduction in active neuron ratio and found that the regression is significant for the WT-light and hAPP-dark groups (Figure S8E). Surprisingly, for the WT-dark group, active neuron ratio reduction is not a significant predictor of AUC ratio reduction (Figure S8E). To verify that it is not an artifact of placing a threshold for activity, we compared the fraction of neurons with different response (dF/F0) levels without placing an activity threshold (Figure S8F). The fraction of highly responsive neurons (>15% dF/F0, which is the average dF/F0 of the top 20% of neurons for a novel grating stimulus) during the first 10 s of stimulus exposure is slightly elevated in WT-dark mice (Figure S8F) but their post-training levels were similar to the pre-training WT-light group (Figure S8F). Furthermore, training-associated reduction of high-responsive neurons is not significant in WT-dark mice (Figure 5E). This suggests that dark housing increases the excitability of neurons to a visual stimulus. The results from other groups were consistent with previous results; the fraction of high-responsive neurons did not reduce in hAPP-light mice after training, whereas WT-light (post/pre ratio: 0.45) and hAPP-dark (post/pre ratio: 0.15) mice showed a reduction (Figure 5E).

When a population of neurons is more excitable, longer-duration activity may cause a more persistent stimulus representation. We next tested how the stability of a neural representation (persistence) to a stimulus was influenced by familiarity and whether the duration changed under hyperexcitable conditions. To do this, we calculated how similar the neural population activity was between pairs of imaging frames of various lags (STAR Methods). A value of 1 indicates complete overlap between frames with the same combination of neurons active, while a value of 0 occurs when the activity is orthogonal, a completely different combination of active neurons. We examined the imaging frames during the first 10 s of the stimulus and measured the decay rate (τ) of the neurons’ similarity or overlap with future activity. More negative values represent faster decay (less persistence) or change of the neural representation. A larger post/pre ratio indicates that training made the neural representation less persistent. We found that WT-light mice showed faster decay of neural overlap (post/pre ratio: 1.44; Figure 5F) after training, suggesting that neural representation is more transient with familiarity. Interestingly, the pre-training decay of neural overlap was slightly slower in the WT-dark group, and their post-training levels were similar to the pre-training WT-light group (Figure 5F). Conversely, for hAPP mice, training did not alter the rate of decay under the light condition, whereas the dark group showed increased decay with training (post/pre ratio: 1.94; Figure 5F). These results show that training induced more transient neural representations in the WT-light and the hAPP-dark groups. In hyperexcitable states (WT-dark and hAPP-light groups), however, the neural representation did not change with training to repeated stimuli but, rather, persisted at the pre-training levels.

The increase in visual stimulus-evoked excitability in dark-housed WT mice could result from disinhibition,^59,60^ elicited by visual deprivation, to maintain activity homeostasis. We have shown previously that excitatory synapses become less dynamic in the dark at the structural level.^43^ Here, we found that the ratio of gain/loss of inhibitory synapses is significantly reduced in the visual cortex when mice were housed in the dark compared with light, indicating that deprivation-associated disinhibition manifests at the structural level (Figures 5G and 5H).

### Non-specific visual experiences differentially modulate the behavioral expression of visual familiarity in WT and hAPP mice

Repeated experience of a stimulus results in visual recognition memory, expressed behaviorally as a reduced exploration of the stimulus.^33^ If any of the network correlates for familiarity influence the exploration of the stimulus, then we expected that non-specific visual experiences would influence the behavioral expression of visual familiarity.

**Figure 6.**
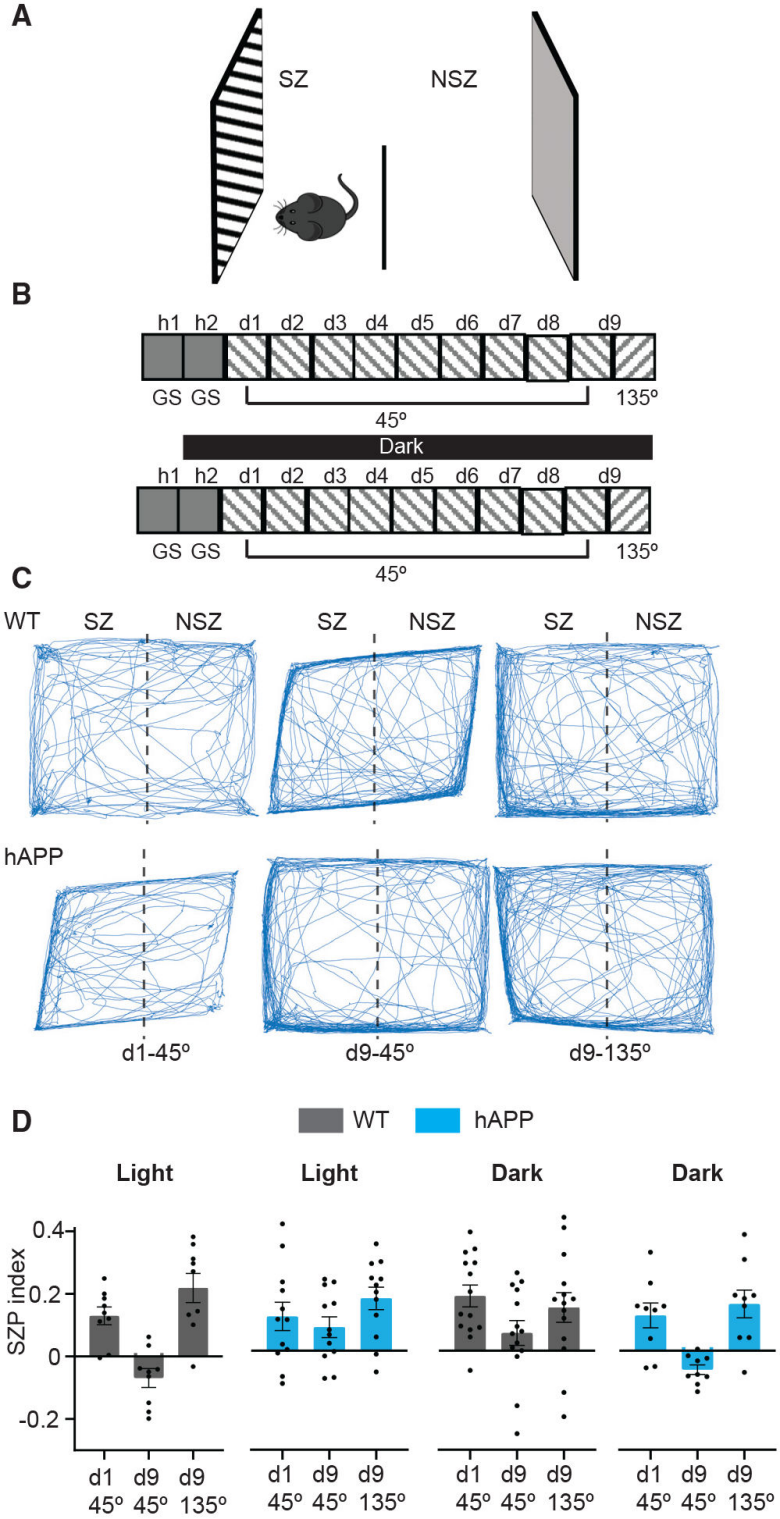
Other visual experiences differentially influence visual recognition memory (A) Representation of a mouse in a chamber with two identical monitors. The arena is divided into two equal zones: stimulus zone (SZ) and nonstimulus zone (NSZ). (B) Timeline for the paradigm (dark, bottom: dark housing). Days and stimuli are indicated. h1 and h2 are habituation days. GS, gray screen. (C) Representative traces of a WT (top) and hAPP (bottom) mouse’s position during the five blocks of stimulus on the first day of 45° stimulus (day 1) and on the test day (day 9) with 45° or 135° stimuli. (D) Stimulus zone preference (SZP) index for hAPP mice and WT sibling controls. Light and dark refer to mice housed in the normal light cycle (12 h light:12 h dark) and complete darkness. n = 9 WT-light, 12 hAPP-light, 14 WT-dark, and 9 hAPP-dark mice. Data are mean ± SEM. p < 0.05 for training × genotype × light condition interaction using three-way repeated-measures ANOVA. Selected multiple comparisons are presented in Table S1. Circles in the histogram represent individual mouse values. See also Figures S9 and S10.

We measured behavioral habituation for the repeatedly experienced 45° phase-reversing grating stimulus in WT and hAPP mice with separate groups of mice housed in light or dark. Following habituation to the apparatus, the stimulus was presented for 8 days in one of the two randomly chosen monitors placed on two sides of the square chamber; the other displayed a gray screen (Figures 6A and 6B). On the following test day, mice were presented with the same 45° stimulus and a novel 135° stimulus (control for motivation to explore). The stimulus zone preference (SZP) index was calculated as the difference in the time mice spent exploring the stimulus and non-stimulus zones divided by the total time. More positive values indicate that the mice spent more time in the stimulus zone. On the first day, all groups of mice had a similar positive SZP index, indicating a preference for stimulus exploration (Figures 6C and 6D; Table S1). We confirmed that the mice explored novel stimuli regardless of the side of presentation to rule out any spatial bias for exploration (Figure S9A). On the test day, WT-light and hAPP-dark mice had a negative SZP index for the 45° stimulus and a positive SZP index for the novel 135° stimulus, indicating that they formed a visual recognition memory (Figures 6C and 6D, Table S1). The total movement in the chamber increased over the days but was similar between the genotypes (Figure S9B). Interestingly, on the test day, the SZP index for the 45° stimulus was not significantly different from 135° for WT-dark and hAPP-light mice, suggesting that these mice are deficient in visual recognition memory (Figures 6C and 6D; Table S1). These results show that non-specific visual experiences improve visual recognition memory of a specific stimulus under non-pathological conditions but disrupt it in amyloidosis.

## DISCUSSION

### E/I imbalance in amyloidosis

We show that hyperactivity in amyloidosis is rooted in the anatomy of cortical neurons. We found that apical dendrites of visual cortical neurons in pre-plaque hAPP mice have a reduced range of excitatory synapse densities because of a decreased fraction of dendrites with high and low synapse densities (Figure 1D). A reduced fraction of low-density dendrites suggests that amyloid increases excitatory synapse density, which would increase neuronal activity and curtail further increases in synapse densities, leading to a narrower range of densities.

An increase in the E/I ratio more selectively in the apical dendrites could be due to intrinsic biological differences between apical and basal dendrites. In such a scenario, the observed differences may broadly apply to other cortical regions and perhaps the hippocampus. Selective apical dendrite vulnerability is reported in amyloid mouse models^61^ and patients with AD.^62^ However, numerous reports showed apical and basal dendrite vulnerability in amyloidosis. Alternatively, the differences in apical and basal dendrites could be due to differential presynaptic innervation. The vulnerability of apical and basal dendrites may depend on circuit properties and may not be generalizable across the cortex or the brain. Consistent with this idea, excitability properties of cortical regions appear to be different in amyloid models. In contrast to hyperactivity in the visual cortex, the parietal cortex has reduced glutamatergic activity.^17,63^ Similarly, the somatosensory cortex also shows reduced activity.^64^ In the visual cortex, feedforward inputs primarily target the basal dendrites, whereas the feedback inputs innervate apical dendrites.^65^ Feedback inputs to the visual cortex could be more vulnerable to high-amyloid conditions. Alternatively, changes in feedback inputs to apical dendrites may compensate for a possible amyloid-induced increase in somatic inhibition by parvalbumin neurons^66,67^ not captured by our *in vivo* imaging approach. The increased orientation selectivity could partly stem from increased inhibition.^68^

Decreased PSD95 but normal synaptophysin puncta levels in CA1 have been reported previously.^69^ In contrast, reduced synaptophysin levels have also been observed in the same model.^70^ Similarly, normal spine densities in the CA1 region of J20 mice at the same age have also been reported.^71^ Interestingly, an early increase in excitatory synaptic density in CA1 has been reported in a different amyloid model.^47^ These results indicate that structural synaptic alterations in apical and basal dendrites may depend on the age, circuit, strain, and, perhaps, sub-strain in amyloidosis. Low levels of amyloid promote long-term potentiation (LTP) but disrupt it at higher concentrations.^72^ Therefore, the differences in amyloid levels may determine how amyloid influences excitatory synapse density.

The role of amyloid in regulating inhibitory synapses is not conclusive, with studies showing increased, reduced, or little change in their density or activity.^8,13,16,17,20,66,73-77^ Here, we see a slight but not significant reduction in inhibitory synapse density in hAPP mice. The ratio of these two synapse types is more consequential to neuronal activity than their densities. Thus, imaging both synapse types in the same dendrites allowed us to determine a structural basis for neuronal hyperactivity. Our findings also reveal that gross structural abnormalities are not a prerequisite for memory deficits, and subtle changes in multiple brain regions, including the visual cortex, may contribute to disease progression.

### Neuronal hyperactivity and circuit architecture

Under non-pathological conditions, energy efficiency is achieved by sparsifying neuronal activity, with few high-responsive neurons encoding a stimulus.^50^ Multiple mechanisms may contribute to the sparse activity, including inhibition and decreased intrinsic excitability.^78,79^ Therefore, increased excitability would be expected to decrease population sparseness. However, we found that hyperactivity in amyloidosis increases population sparseness. How could increased excitability also increase population sparseness? Given energy constrains in the brain, we speculate that increased population sparseness is a compensatory adaptation for the higher energy demand associated with hyperactivity. Low-responding neurons reduce their activity to compensate for the hyperactivity of a few high-responding neurons so that population response amplitude is maintained. These findings are consistent with prior reports of hyperactive and hypoactive neurons near amyloid plaques.^80,81^ However, this leads to increased population sparseness, decreased functional connectivity, and increased memory interference. These results also argue that disrupted functional connectivity is more likely to be a consequence than a cause for AD pathologies.

Increased cFos+ cell density contrasts with population sparseness observed during calcium imaging. We speculate that two factors may contribute to the observation. One is that cFos expression in weakly responsive neurons could be below the threshold of detection, and their reduction in hAPP mice does not reduce the cFos counts. Second, each spiking event has a higher amplitude in hAPP mice, and the resultant higher calcium influx may contribute to elevated cFos expression beyond the threshold used for identifying c-Fos+ neurons.

### A model for the role of E/I balance in regulating visual recognition memory

Multiple network correlates of visual familiarity in the visual cortex were revealed in WT-light mice: reduced population response amplitude, fraction of high-responding neurons, functional connectivity, persistence of neural representation, and increased population sparsification. All of these familiarity correlates were disrupted in hAPP mice because of interference from non-specific visual experiences. Surprisingly, two of these correlates, the reduced fraction of high-responsive neurons and persistence of neural representation, in post-training WT-dark mice were also disrupted, indicating that non-specific visual experiences are required for some of the familiarity correlates in WT mice.

The conditions (WT-dark, hAPP-light) that failed to reduce the persistence of neural representation and constrain the fraction of high-responsive neurons as a stimulus became familiar had impaired behavioral expression of visual familiarity. Therefore, we propose that constraining neural activity persistence and the fraction of high-responsive neurons to a repeatedly experienced stimulus below a threshold serve as a code for visual familiarity (Figure S10).

Hyperactivity and reduced stimulus specificity to natural visual experiences could lead to continual coactivity of the few highly connected neurons in amyloidosis. Intact Hebbian plasticity mechanisms would prevent a reduction in functional connectivity despite repeated exposure to grating stimuli. Intact connectivity would keep the fraction of high-responsive neurons and the persistence of neural activity above a threshold for visual familiarity (Figure S10). Coactivity of a higher fraction of high-responsive neurons and longer persistence of their activity would promote behavioral exploration of the stimulus (Figure S10). Contrary to the commonly held view that synapse loss and impaired Hebbian plasticity mechanisms underlie memory deficits in AD, we propose that memory deficits may also emerge as a result of intact Hebbian plasticity mechanisms and unaltered synaptic density because of E/I imbalance.

Under non-pathological conditions, visual recognition memory deficit arises in the absence of other visual experiences despite normal plasticity (reduced evoked AUC, functional connectivity, and increased population sparseness). This is surprising because the memory is expected to be more robust when experiencing only one type of visual stimulus. However, memory-irrelevant visual experience constrains the fraction of high-responsive neurons to memory-relevant stimulus and reduces the persistence of stimulus representation. In the absence of non-specific experiences, increased excitability may interfere with these familiarity representations and lead to memory deficits. Alternatively, continued exploration of a stimulus in the dark could be mediated by top-down or neuromodulatory mechanisms. Our findings suggest that the absolute fraction of high-responsive neurons and the duration of the neural representation of the stimulus, rather than relative plasticity, are relevant for visual recognition memory. The cellular mechanisms of visual habituation may vary depending on the brain region or the stimulus specificity of habituation. In layer 4 neurons of the visual cortex, repeated stimulus exposure led to the depression of cellular response, but unlike layer 2/3 neurons,^56^ it did not reduce the fraction of active neurons responding to the familiar stimulus.^57^ In addition, an increase in spontaneous activity rather than a reduction in evoked activity was observed in layer 2/3 neurons, but the habituation was not specific to the familiar stimulus.^34^

### Limitations of the study

*In vivo* dendritic imaging does not capture all inhibitory synapses, such as the soma targeting parvalbumin neurons,^82^ and changes to ion channel composition. Furthermore, the mouse model used in this study overexpresses amyloid precursor protein with familial AD mutations,^70^ and whether non-overexpressing mice show similar phenotypes remains to be tested. APP knockin mice (AppNL-G-F) with familial AD mutations also show hyperexcitability.^83^ The amyloid levels in knockin (KI) mice are higher than that observed in J20 mice, but hyperexcitability is slightly lower, indicating that other factors may exacerbate hyperexcitability. Finally, whether memory interference driven by hyperexcitability is limited to the visual cortex remains to be tested, although interference has been associated with impaired recall of a hippocampus-dependent task in another amyloid model.^84^

## STAR★METHODS

### RESOURCE AVAILABILITY

#### Lead contact

Further information and requests for resources and reagents should be directed to and will be fulfilled by the lead contact, Jai Subramanian (jaichandar@ku.edu).

#### Materials availability

This study did not generate unique reagents.

#### Data and code availability

- Data generated in this study are available from the lead contact upon request
- All original code used in this study for synaptic puncta detection, intrinsic signal analysis, and spectral unmixing has been deposited at Zenodo and is publicly available as of the date of publication. DOIs are listed in the key resource table.
- Any additional information required to reanalyze the data reported in this work paper is available from the lead contact upon request.

### EXPERIMENTAL MODEL AND SUBJECT DETAILS

#### Mice

All animal procedures are approved by the University of Kansas Institute of Animal Use and Care Committee and meet the NIH guidelines for the use and care of vertebrate animals. PDGF-hAPP transgenic mice (J20 line; Gladstone) were maintained as heterozygotes for the hAPP transgene by breeding heterozygous J20 male mice with WT female mice. J20-GCaMP6s mice were generated by breeding J20 male mice with female C57BL/6J-Tg (Thy1-GCaMP6s) GP4.3Dkim/J (Strain: 024275, JAX). A maximum of five mice were housed in a standard cage but individually housed after the cranial window surgery. Mice were housed on a 12h-light/12h-dark cycle except for the group that went through a period of visual deprivation (24h-dark).

#### Cell culture

SH-SY5Y cells (American Type Culture Collection), a neuroblastoma cell line originally derived from a human female, were grown in Eagle’s Minimum Essential Medium supplemented with 5% fetal bovie serum at 37°C, 5% v/v CO_2_, and 95% humidity.

### METHOD DETAILS

#### DNA constructs

The Cre dependent TdTomato (pFudioTdTomatoW), Teal-gephyrin (pFudioTealgephyrinW) and PSD95-venus (pFudioPSD95venusW) plasmids are a kind gift from Dr. Elly Nedivi. Cre recombinase is expressed from pSIN-W-PGK-Cre plasmid.^86^ The combination of pSIN-W-PGK-Cre and pFUGW based fluorescently labeled gephyrin and PSD95 synaptic markers has been shown to reliably represent inhibitory and excitatory synapses, respectively.^44,85^

#### In utero electroporation (IUE)

Timed pregnant matings were set between heterozygous J20 males and WT females of the same genetic background. Half of the litter were heterozygous for APP mutations, and the other half were WT (control). E15.5–16.5 embryos received ~3μg of plasmids in 1μL Tris-EDTA (1:1:0.5:0.15 M ratio of pFudioTdTomatoW, pFudioTealgephyrinW, pFudioPSD95venusW, and pSIN-W-PGK-Cre, respectively) mixed with 0.1% fast green into the right lateral ventricle using a 32-gauge Hamilton Syringe (Hamilton company). A pair of platinum electrodes (Protech International) placed to target the visual cortex was used to provide five pulses of 36 V (50 ms duration at 1 Hz) from a square wave electroporator (ECM830, Harvard Apparatus).

#### Cranial window

4-6-month-old J20 and WT mice received a cranial window over the visual cortex on the right hemisphere. A small scalp incision was made over the midline of the skull. Soft tissues were reflected laterally by blunt dissection, and the pericranium was gently scraped. A 5-mm diameter circle covering the visual cortex was scored using a biopsy punch. The skull was thinned along the scored circles with a fine drill using a sterile 0.5mm diameter round burr (Fine Science Tools). The bone flap was carefully removed with fine forceps leaving behind the dura. A 5-mm diameter sterile circular glass coverslip (Harvard Apparatus) was positioned over the openings. Vetbond was applied over the juncture between coverslip and bone as firm pressure was used to keep the coverslips in place. Metabond (C&B Metabond) was applied over the exposed skull. ~2-weeks after the surgery, a titanium head-post was affixed around the window to restrain mice during imaging. For GCaMP6s expressing mice, a light-blocking cone was attached to the titanium headpost to block monitor light from reaching the PMTs during imaging of visually evoked activity.

#### Optical intrinsic signal imaging

Optical intrinsic signal imaging was performed 14 days after cranial window surgeries to map the location of the visual cortex.^44,87^ Imaging was performed in a custom-built upright microscope with a 4× objective (Nikon). Lightly anesthetized mice were positioned 20 cm in front of a high refresh rate monitor displaying a horizontal bar (1° of the visual field) drifting at 10 Hz. Images were collected using an sCMOS camera at 5Hz (1024 × 1024 pixels; Photometrics). A fiber-coupled LED, powered by T-Cube LED drivers (Thorlabs), was used to deliver 610 nm light to illuminate the cortex (500–600 μm below the dura). Reference vasculature was imaged with a 470 nm light. Images were downsized to 256 × 256 pixels, and cortical intrinsic signals were computed by extracting the Fourier component of light reflectance changes to matched stimulus frequency. The fractional change in reflectance represents response magnitude, and the magnitude maps were thresholded at 30% of the peak-response amplitude. The visual cortex was mapped by overlaying the magnitude maps over the 470nm reference image.

#### Widefield calcium imaging

For GCaMP6s expressing mice, widefield calcium imaging instead of intrinsic signal imaging was used to map the location of the visual cortex. The mapping protocol was similar to intrinsic signal imaging, except that fluorescence was imaged rather than reflected light. GCaMP6 was excited by an LED (Lambda FLED, Sutter) filtered through a bandpass filter (470/40, 49,002 Chroma), and the emission was filtered through a 525/50 bandpass filter.

#### Two-photon imaging

For synaptic structural imaging, anesthetized mice with sparsely labeled neurons within the mapped visual cortex were imaged using a Sutter MOM multiphoton microscope. The Ti: sapphire laser (MaiTai HP: Newport SpectraPhysics; 915 nm) was routed to the microscope using table optics. The power was adjusted using a rotating half-wave plate and a polarizing beam splitter. A pair of galvanometric mirrors scan the laser beams to the back aperture of the objective (Nikon 16 × 0.8 NA). The output power from the objective was set to 40-50mW. Emission signal was collected through the same objective, passed through a short pass filter to block infrared wavelengths, and routed to three GaASP PMTs after passing through appropriate bandpass filters (488/50, 540/50, and 617/73 for Teal, YFP, and TdTomato fluorescence, respectively). Image acquisition was controlled by Scanimage (Vidrio Technologies), and images were obtained at 0.16Hz. The imaging field covered 133 × 133x~150 μm (1024 × 1024 XY pixels, Z step - 1 μm). For GCaMP6 imaging, neurons within the mapped visual cortex (~100–150 μm below the dura) were imaged at 4.22 Hz in head-restrained awake mice restrained in a body tube. The excitation wavelength was set to 940 nm, and the power was adjusted (20-40mW) to avoid signal saturation. The imaging field was a single Z frame of 336 × 336 μm (256 × 256 pixels) consisting of ~50–100 cells.

#### Visual stimulus to head restrained mice

Visual stimuli were delivered on a high refresh rate monitor placed 20 cm in front of the head restrained animals covering 94° × 61° of the visual field. The software for generating visual stimuli was modified from a custom written stimulus suite (a kind gift from Dr. Mark Bear’s lab) written in MATLAB (Mathworks) using the PsychToolbox extension. Mice were habituated to a gray screen by head-restraining them under the microscope for two days (30 min each day). For measuring the neuronal activity and functional connectivity, visual stimuli consisted of 30 s of the gray screen followed by 8-cycles of 100% contrast, sinusoidal, phase reversing (2 Hz, 0.05 cycles/degree) grating stimuli of different orientations (0°, 45°, 90°, 135° - 3 s each) and two sets of ten natural images (0.3 s/image–3 s per set) interspersed with 6 s of the gray screen. The order of stimuli was different in each cycle. For a couple of mice, only one set of natural images was used. Grayscale natural images were obtained from Berkeley Segmentation Dataset, contrast normalized and resized to 1600 × 1068 pixels. Grating stimuli covered the entire monitor display value range between black and white. Gamma correction was performed to ensure the total luminance in the gray screen and grating stimuli were the same.

For plasticity experiments, mice were habituated as above for two days. For the next seven days, head restrained mice were exposed to two sessions of 60 s gray screen followed by five blocks of 100 s of phase reversing grating stimulus with 30 s of the gray screen between blocks. The two sessions were separated by ~1–2 h. On the first session of the first day of stimulus (pre-training), 10–15 min after head-fixation GCaMP6 response was first imaged without visual stimulus (total darkness) to record spontaneous activity. Five minutes later, imaging was performed during the first 60 s of the gray screen and the first block of 45° grating stimulus. We tried to closely match the same field of view imaged in the first session for post-training imaging on the seventh day. Due to slight differences in Z positioning, a part of the neuronal population was identical between sessions. To confirm that any reduction in neuronal activity is not due to poor imaging conditions or the animal’s behavioral state on that day, we also recorded the response to 60 s gray screen and 100 s of a novel (135°) stimulus 15 min after the end of the first session. The mice were then housed in a dark room (24-h dark cycle) for one day, and the process was repeated, except that mice were repeatedly exposed to 75° instead of 45°. On the final day, a novel 165° stimulus was used as a control for imaging quality. During these experiments, care was taken to avoid light exposure other than the visual stimulus.

#### Free moving behavior

We used a visual recognition memory paradigm for oriented grating stimulus.^33^ In this paradigm, mice were habituated for two days (30 min each) to a chamber with two identical monitors displaying a gray screen on either side. For the next eight days, mice explored the chamber for 2 × 15 min sessions, with each session separated by 1–2 h. The gray screen was presented on both monitors for the first 5 min. This was followed by five blocks of 100% contrast sinusoidal grating of a specific orientation (45°) that phase reverse at 2 Hz with a 30-s inter-block interval in one of the randomly chosen monitors. The other monitor continued to remain gray. On the test day, mice were presented with the now familiar 45° and a novel 135° stimulus in separate sessions. The position of mice in the apparatus was tracked using a force-plate actometer with a square sensing surface measuring 42 cm on each side. Force samples from each of the four force transducers that support the sensing surface were taken at a rate of 100 samples/s via a USB-based data acquisition device controlled by a computer running custom-written Visual Basic software. The side of the stimulus presentation was tracked using a camera.

#### Tissue preparation and immunohistochemistry

Mouse cages were brought to the surgical suite at least 5 h before brain extractions to avoid capturing c-Fos expression elicited by movement or contextual novelty. ~5-month-old (for c-Fos and 6e10 analysis) or 3.5–5 month old (for synaptic puncta analysis) J20 and WT mice were deeply anesthetized by intraperitoneal injection of 2% avertin in phosphate-buffered saline (PBS), pH 7.4,and transcardially perfused with cold PBS followed by 4% paraformaldehyde. The brains were extracted and post-fixed in 4% PFA overnight at 4°C, followed by storage in PBS. For c-Fos/6e10 analysis, the brains were embedded in 4% oxidized agarose^88^ to limit artifacts during sectioning. Blocks were then cut into 40μm thick coronal slices on a microtome (Leica VT1000 S). For synaptic puncta analysis, the brains were then cryoprotected overnight at 4°C in 15% (w/v) and then in 30% (w/v) sucrose in phosphate buffer (PB). The brains were sectioned coronally at 20 μm thickness on a microtome and collected in PBS with sodium azide (0.02%).

3-4 evenly spaced slices spanning the primary visual cortex for each brain were fluorescently immunolabeled for c-Fos and amyloid. Sections were permeabilized for 2h at room temperature in a 1% Triton X-100 and 10% normal goat serum (NGS) solution in PBS followed by incubation with mouse clone-6E10 antibody (1:250, BioLegend) and rabbit anti-c-Fos (1:1000, CST) in a PBS solution containing 0.1% Triton X-100 and 5% NGS overnight at 4°C. Sections were then washed 3X with PBS and incubated with Alexa 647-conjugated goat anti-mouse antibody (1:2000; Fisher) and Alexa 555-conjugated goat anti-rabbit antibody (1:2000; Fisher) for 2 h in a PBS solution containing 0.1% Triton X-100 and 5% NGS at room temperature, followed by three washes with PBS before mounting on glass slides. Slices were imaged using an ImageXpress Pico automated imaging system (Molecular Devices, San Jose, CA) with a 10× objective (Leica HC PL FLUOTAR 10×/0.32).

For synaptic puncta immunohistochemistry, the brain sections containing both hippocampus and visual cortex (bregma: −3.68mm to −2.78mm) were immersed in 10mM sodium citrate solution and kept in boiling water for 5 min for antigen retrieval. The brain sections were let to cool down and permeabilized for 2 h at room temperature in a 1% Triton X-100 and 10% normal goat serum (NGS) solution in PBS, followed by incubation with mouse anti-PSD-95 (1:1000, Thermo Fisher Scientific) and rabbit anti-gephyrin (1:1000, Synaptic Systems) in a PBS solution containing 0.2% Triton X-100 and 5% NGS overnight at 4°C. Sections were then washed 3X with PBS and incubated with Alexa 647-conjugated goat anti-rabbit antibody (1:2000; Fisher) and Alexa 555-conjugated goat anti-mouse antibody (1:2000; Fisher) for 2 h in a PBS solution containing 0.2% Triton X-100 and 5% NGS at room temperature, followed by three washes with PBS before mounting on glass slides. ~175 μm × 175 μm images from two slices per mouse, containing layers 1 and 2 of the visual cortex, striatum oriens (SO), and striatum radiatum (SR) of the hippocampus, were imaged on a laser scanning confocal microscope (Leica, DM6-Q model; performed at Microscopy and Analytical Imaging Research Resource Core Laboratory), using a 63x (NA 1.30) objective. Alexa 555 and 647 were excited with 561 nm, and 635 nm lasers, respectively, and the emission was collected on a 12-bit spectral PMT detector.

### QUANTIFICATION AND STATISTICAL ANALYSIS

#### *In vivo* synaptic imaging analysis

The signal collected in each PMT is a combination of signals from the three fluorophores (Teal, Venus, and TdTomato) due to their overlapping emission spectra. The relative contribution of signal from each fluorophore to each PMT was calculated by imaging SH-SY5Y cells expressing single fluorophores. We used spectral linear unmixing to reassign the signal from each fluorophore to the appropriate PMT.^44^ To normalize the signal relative to local dendritic volume, we normalized the fluorescence in the synaptic channels to that of the cell fill channel.^89^ A normalization factor was calculated as the ratio of the mean pixel value of a chosen dendrite in the cell fill channel to the synaptic channel. Each pixel value in the synaptic channel was then multiplied by the normalization factor, and the pixel value of the cell fill channel was subtracted on a pixel-to-pixel basis.

Synaptic puncta were identified in multiple steps. For the initial automated identification of PSD95 and gephyrin puncta, we created a plugin for FIJI^90^ from a combination of plugins previously available. Sections of dendrites were traced using the Simple Neurite Tracer plugin to create a binary image stack of the trace. The trace was dilated using the Dilate 3D plugin,^90^ resulting in a 3D binary image used to mask the original image. A custom radius was entered to determine the thickness of the mask from the center of the branch in the XY plane, and a second radius determined the thickness in the z axis. The resulting masked image was split into three separate channels, each marked with different fluorescent proteins. Contrast enhancement was performed in each channel containing puncta.^90^ Background subtraction was then performed using a user-entered rolling ball radius on each image in the stack. A local threshold was applied to each slice in the image stack. The threshold value at each pixel was calculated as a bias added to the median value of the surrounding pixels within a customizable radius. The plugin 3D watershed split^91^ was used on each resulting binary image to separate groups of overlapping puncta. The plugin 3D Object Counter^92^ was used to analyze the binary images and find the positions and size of each puncta. The 3d Object Counter plugin results were exported as files in the .csv format. A macro was used to read the coordinates of the detected objects from the.csv files and place corresponding markers in the first linked image of a custom-written 4D point tracking system implemented in Fiji using a modified version of the ObjectJ plugin.^44^ After the markers were placed, the IDs of the auto-detected objects were saved in the ObjectJ file.

The detected PSD95 and gephyrin puncta were verified for accuracy, and missed puncta were manually added. Gephyrin puncta and PSD95 puncta were scored as synapses if they were present in two consecutive frames and that they consisted of at least 8–9 clustered pixels or 4–5 clustered pixels, respectively. Excitatory synapses emanating perpendicular to the shaft were not included in the analysis. The synaptic density in non-transgenic mice is comparable to previously published values.^43,44^ Two investigators proofread the synapse count, and one of them was blind to the genotype. Fractional gain of inhibitory synapses between two sessions S1 and S2, was calculated as the number of new gephyrin puncta in S2 divided by the total number of gephyrin puncta in S2. The fractional loss of inhibitory synapses was calculated as gephyrin puncta lost in S2 divided by the total number of gephyrin puncta in S1.

A total of 5251 (WT), 6250 (hAPP) PSD95^+^, 1064 (WT), 1301 (hAPP) PSD95^−^, 1190 (WT), 1268 (hAPP) PSD95^+^ and gephyrin^+^ spines, and 1944 (WT), 1965 (hAPP) inhibitory shaft synapses were counted from 64 (WT), 73(hAPP) apical (originating from the apex of soma) and 77 (WT); 79(hAPP) basal dendrites from 11 (7 males and 4 females; WT) and 12 (7 males and 5 females hAPP) mice for E/I balance experiments. Gephyrin dynamics was calculated from ~1000 inhibitory synapses from 6 cells (5 WT mice - 2 females and 3 males).

#### Calcium imaging analysis

Motion registration and ROI detection in the time-series images were performed using Suite2p.^93^ Tau and neuropil coefficient for spike deconvolution were set at 2.0 and 0.5, respectively. Suite2p generated ROIs were chosen as cells (cellular ROI) if the soma was visible in the mean or maximum projection image. Cellular fluorescence (F) was corrected for neuropil contamination, estimated as the ratio of blood vessel fluorescence to that of neuropil (Fneu). The value ranged from ~0.3–0.7, and we used 0.5 as the correction factor. Neuropil corrected fluorescence (Fcorr) is calculated as F - (0.5xFneu). Cellular ROIs that did not have at least one peak greater than 10% dF/F0, calculated as (Fcorr – F0)/F0, where F0 is defined as the mode of the Fcorr density distribution,^94^ anywhere in the time series were excluded. The 10% dF/F0 could lie anywhere in the time series (corresponding to the gray screen or the stimulus period) and is averaged out by the variation between trials.

To analyze spike event frequency and amplitude for spontaneous and stimulus evoked activity, deconvolved spikes obtained from Suite2p for cellular ROIs were thresholded (>2 SD from the mean to remove noise artifacts). The frequency of spike events is calculated as the number of identified spike events/defined time period (100 s for Figures 3A-3D).

For experiments with multiple grating and natural images stimuli, dF/F0 for each stimulus from all the cycles (trials) were averaged for all neurons. Neurons are considered active if the trial-averaged dF/F0 of the 3 s stimulus period is greater than 5% (high-threshold) or two standard deviations of baseline dF/F0 (low-threshold) and is different from the 3 s of gray screen preceding the stimulus (p < 0.05, paired t test). Mean dF/F0 for a stimulus was calculated by averaging all active neurons’ mean dF/F0. The area under the curve (AUC) for the dF/F0 response is calculated using the trapz function in MATLAB. The fraction of active (visually responsive) neurons was calculated as the number of neurons active for at least one stimulus divided by the number of neurons identified by Suite2p.

The MATLAB-based GUI for ensemble identification was a kind gift from Drs. Jesus Perez Ortega and Rafael Yuste.^52^ Deconvolved spikes obtained from Suite2p for cellular ROIs were thresholded (>2 SD from the mean) and binarized. Briefly, neuron pairs are considered functionally connected if the number of their coactive frames exceeds 95% of the cumulative probability distribution generated by a 1000 random circular shift of their activity. All imaging frames (vectors) that do not contain at least three coactive neurons were excluded. Hierarchical clustering using simple linkage was used to identify vectors with greater than 50% Jaccard similarity, and non-similar vectors were excluded from the raster. More similar vectors were clustered with Ward linkage and grouped based on contrast index.

To identify neurons participating in an ensemble, Pearson correlation coefficient for the coactivity of neurons and an ensemble was calculated. A binary vector Vj representing the imaging frames when the ensemble was active (1) or not (0) was generated. The correlation of neurons *a* and *b* activities with ensemble j activity would be Pj,a and Pj,b, respectively. To assess if neurons *a* and *b* are functionally connected within ensemble *j*, an ensemble weight (Wj,ab) was calculated as Pj,a. Pj,b. Coab, where Coab is the correlation of neurons *a* and *b* activities. To test the significance, 1000 surrogates were obtained by shuffling neurons *a* and *b* activities and used the correlation from each iteration to calculate a surrogate weight (SWj,ab,i). If Wj,ab is greater than 95% of the cumulative probability distribution of surrogate weights, then a functional connection was placed between neurons *a* and *b* within ensemble *j*. A neuron will be part of the ensemble if it has at least one functional connection.

The functional connectivity matrix generated above was used to determine the node degree - the number of edges connected to each node (neuron) during the entire imaging period for Figure 4B or during the first 10 s of the grating stimuli (Figures 5D and S8C) using MATLAB graph and degree functions. The skew function was used to calculate the skewness of degree distribution.

Population sparseness is calculated as

$$1-\frac{\left(\Sigma iRi∕n)2∕(\Sigma iRi2∕n\right)}{\left(1-1∕n\right)}$$

where Ri is the AUC of the trial-averaged dF/F0 response during the 3-s stimulus (for the multi-stimuli experiment) and 10-s stimulus (for plasticity experiment), and n is the number of identified neurons. The selectivity index for natural images in neurons considered active for natural images is calculated as 1 − (*∑iRi /n*)2/(*∑iRi*2 /*n*), where *Ri* is the amplitude of trial-averaged (4 cycles of the same set of natural images) deconvolved spike event in individual imaging frames during the 3 s natural image stimulus period encompassing 13 (n) imaging frames. We used deconvolved spikes to avoid confounds from the slow decay kinetics of calcium transients.

To obtain an orientation tuning curve, the trial-averaged AUC of dF/F0 during the 3 s of each grating stimulus was fit as a function of stimulus angle (φ) with a von Mises function (Equation 1) in neurons responsive to any grating stimuli.^95,96^

(Equation 1) $$f(\varphi )=Ae^{K(cos[2(\varphi -\theta )]-1)}+b$$

The function is defined by four fit parameters: a preferred stimulus orientation that gives the maximum response (*θ*), a tuning curve width (*K*), a response amplitude (A), and an intercept (b). Fits were calculated with a maximum likelihood estimate of *θ* and *K* of using CircStat in MATLAB,^97^ and least-squares regression was then used to identify A and b. The fraction of explained variance, R^2^, was calculated, and the full width at half-maximum was calculated as FWHM = arccos[ln(1/2eK + 1/2e-K)/K] for fits that accounted for 70% of the variance in the data.

For single orientation stimulus plasticity experiments, F0 was calculated as the mean Fcorr of 10 s preceding the start of the stimulus. All analyses were limited to response during the first 10 s of stimulus exposure. Cellular ROIs with F0 greater or less than two SD from the mean were not included in the analysis. To ensure only good quality recordings were analyzed, we only included mice whose mean population dF/F0 was at least 1% for the novel stimulus (response to the first session with 45° and 135° for light conditions, and 75° and 165° stimulus for the dark conditions). AUC of dF/F0 for the first 10 s of the grating stimulus was measured, and the ratio of AUC_tensec_ of post-training to pre-training was used as a measure for plasticity. Cellular ROIs with greater than 5% mean dF/F0 during the first 10 s of stimulus and are significantly different (p < 0.05, paired t test) from the mean dF/F0 of the 10-s gray screen before the stimulus onset were considered as active neurons.

To assess the stability of the neural representation to a given visual stimulus over time, we calculated the similarity of the neural population’s activity between pairs of imaging frames. We used a neural space representation to quantify the neural population activity of each frame. The neural activity for each imaging frame was represented as a datapoint in the neural space.^98^ The activity level for each identified neuron as measured with dF/F0 was mapped to its own neural dimension. This combination or pattern of active neurons thus defined a vector in the neural space and was normalized to a unit vector for each imaging frame. It’s similarity or overlap with other frames was then calculated by taking the dot product between the neural vectors in methods similar to.^99,100^ The neural overlap was calculated as follows:

$$Overlap=\frac{N_{t1}}{\left\|N_{t1}\right\|}•\frac{N_{t2}}{\left\|N_{t2}\right\|}$$

where N_ti_ is the neural vector for a given time, t1, and a time, t2, in the future. Each neural vector is normalized by its magnitude (∥*N_ti_*∥). A value of 1 indicates complete overlap between frames with the same combination of neurons active, while a value of 0 occurs when the activity is orthogonal, a completely different combination of neurons is active. Analysis was performed on all imaging frames within the time window from immediately after stimuli onset until 10 s after onset. Each analysis frame was examined by calculating its overlap with subsequent images with delays of 1–50 frames in the future by calculating the overlap. The overlap values were then averaged for a given delay for all analysis frames in the 10 s window.

The amount of neural overlap as a function of time lag was fit with exponential decay of the following form:

$$Overlap\;=\;(1-b)e^{\tau \cdot t}+b$$

Here, τ defines the rate of decay starting from an Overlap of 1 at no time delay (t = 0), and b represents the final average overlap after a sufficiently long interval. The rate of decay, τ, is always negative, with more negative values representing faster decay corresponding to less persistence or stability in the neural representation during stimulus presentation.

For multiple stimuli experiments, 8 WT (6 males and 2 females) and 6 hAPP (5 males and 1 female) mice were analyzed. ~70–90 ROIS were identified per mouse. For plasticity experiments, 11 WT (4 males and 7 females) and 9 hAPP (5 males and 4 females) mice were analyzed. ~60 ROIs were identified per mouse. Suite2p analysis was performed blind to genotype.

#### Behavior analysis

The voltage measurements from the four sensors of the force plate were smoothed using a moving average filter and converted into x-y coordinates using the following formula

$$X=\frac{\left(X1\;f1+X2\;f2+X3\;f3+X4\;f4\right)}{\left(f1+f2+f3+f4\right)}$$

$$y=\frac{\left(Y1\;f1+Y2\;f2+Y3\;f3+Y4\;f4\right)}{\left(f1+f2+f3+f4\right)}$$

where (X1, Y1), (X2, Y2), (X3, Y3), and (X4, Y4) are the X and Y coordinates of the fixed positions of the four force sensors located at each corner of the square chamber and f1, f2, f3, f4 are the four forces [96]. The data were then down-sampled from 100 Hz to 2 Hz. The half of the chamber closest to the stimulus monitor is considered the stimulus zone, and the other half is the non-stimulus zone. The stimulus zone preference (SZP) index is calculated as the difference in the time mice spent exploring (active exploration at ≥3 cm/s) the stimulus and non-stimulus zones divided by the total exploration time. More positive values indicate that the mice spent more time in the stimulus zone. Mice that did not respond to stimulus on both the sessions on days with a novel stimulus (first day of stimulus and memory test day) were removed from the analysis. Data from the first session of the stimulus on the first day and test day were compared. 9 (WT - light), 12 (hAPP-light), 14 (WT-dark), and 9 (hAPP-dark) male mice were analyzed.

#### Immunofluorescence analysis for c-Fos, 6e10, and synaptic markers

Slice registration, cell detection, and brain region area measurements were performed using NeuroInfo software (MBF Bioscience, Williston, VT). Slices were first mapped in 3D to the Allen CCF v3 to allow automated cell detection and area measurement by region. Bright circular objects against a darker background were automatically detected using a scale-space analysis of the response to Laplacian of Gaussian (LoG) within the expected range of labeled cell body diameters.^101^ Briefly, cells were filtered out from all identified objects with a user-defined threshold based on the strength of the LoG response within an expected range of cell body diameters. Each respective region’s threshold value was set at the 70th LoG strength percentile of identified objects in that region across all WT slices (LoG threshold = 15, range 0–255). Only objects above this LoG strength threshold were included in the analysis to minimize false positives. Cell density was calculated by dividing the number of cells per region by the area per region across all slices for each brain. 8 hAPP (5 males, 3 females) and 5 WT (3 males, 2 females) were used. Individual cell correlation analysis was performed on fluorescence values of each ROI in both the c-Fos (nuclear fluorescence) and the 6E10 (nonnuclear) were collected in FIJI by automatically drawing a 10-pixel radius ROI around identified c-Fos + cells post-proofreading. For animal correlation, mean 6E10 fluorescence values from the entire visual cortex in the analyzed slices were collected in FIJI and compared with the mean fluorescence of c-Fos + cells. 8 hAPP (4 males, 4 females) mice were used for c-Fos/hAPP correlation analysis.

For synaptic markers, analysis is performed as described above for c-Fos except that a 25μm rolling ball radius background subtraction was first performed in Fiji, and the LoG threshold was 10 for the PSD channel and 4 for the Gephyrin channel, range 0–255. Automatically identified puncta (~0.5–1.5μm) were manually proofread, and the remaining false positive identifications were removed before analysis. 8 WT (6 males, 2 females) and 7 hAPP mice (4 males, 3 females) were analyzed blind to their genotype.

#### Statistical analysis

Data were analyzed using Prism 9 or SPSS. No statistical methods were used to predetermine sample sizes. The sample sizes are comparable to previous literature. Test for normal distribution was done with Kolmogorov-Smirnov test. p < 0.05 was considered statistically significant. Sample sizes are reported in figure legends and methods. Samples are individual mice (for most analyses), cells or dendrites. Blinding of genotype identity is mentioned in methods. Statistical procedures are two-sided and are listed in figure legends. The actual p values, *F* and *t*-values, and degrees of freedom are listed in Table S1. For the data presented as ratios of post/pre training, the pre and post training means are provided in Table S2.

## Supplementary Material

1

2

3

## Figures and Tables

**Table:** KEY RESOURCES TABLE

REAGENT or RESOURCE	SOURCE	IDENTIFIER
Antibodies		
Mouse anti-6E10 antibody	BioLegend	Cat# 803002; RRID: AB_2564654
Rabbit anti-c-Fos antibody	CST	Cat# 2250S; RRID:AB_2247211
Mouse anti-PSD-95	ThermoFisher	Cat# MA1-045; RRID:AB_325399
Rabbit anti-gephyrin	Synaptic Systems	Cat# 147018; RRID:AB_2651176
Alexa 647-conjugated goat anti-rabbit antibody	Fisher Scientific	Cat# PIA32733; RRID: AB_2633282
Alexa 555-conjugated goat anti-mouse antibody	Fisher Scientific	Cat# PIA32727; RRID:AB_2633276
Alexa 647-conjugated goat anti-mouse antibody	ThermoFisher	Cat# A21236; RRID:AB_2535805
Alexa 555-conjugated goat anti-rabbit antibody	ThermoFisher	Cat# PIA32732; RRID:AB_2633281
Bacterial and virus strains		
One Shot STBL3 chemically competent E.coli	ThermoFisher	Cat #C737303
Experimental models: Cell lines		
SH-SY5Y cells	ATCC	CRL-2266
Experimental models: Organisms/strains		
PDGF-hAPP transgenic mice (J20 line)	Gladstone	34836-JAX
C57BL/6J-Tg (Thy1-GCaMP6s) GP4.3Dkim/J	JAX	Strain: 024275
Oligonucleotides		
Primer for J20 genotyping 5′ GACAAGTATCTCGAGACA CCTGGGGATGAG-3′	This paper	N/A
Primer for J20 genotyping 5′AAAGAACTTGTAGGTTGGATTTTCGTAGCC-3′	This paper	N/A
For WT (Connexin gene) 5′CCATAAGTCAGGTGTAAAGGAGC-3′	This paper	N/A
For WT (Connexin gene) 5′GAGCATAAAGACAGTGAAGACGG-3′	This paper	N/A
Recombinant DNA		
pFudioTdTomatoW	A gift from Dr. Elly Nedivi	N/A
pFudioTealgephyrinW	Chen et al.^85^	RRID:Addgene_73918
pFudioPSD95venusW	A gift from Dr. Elly Nedivi	N/A
pSIN-W-PGK-Cre	Subramanian et al.^86^	RRID:Addgene_101242
Software and algorithms		
ScanImage Basic	Vidrio Technologies	https://vidriotechnologies.com/scanimage
StimulusSuite	Modified version of Dr. Mark Bear’s lab version	N/A
Neural_Ensemble_Analysis.m	Gift from Dr. Rafael Yuste lab	N/A
4D point tracking system, ObjectJ	Villa et al.^44^	N/A
Deposited code	This paper	https://zenodo.org/badge/latestdoi/568293797
Cell reporter express (CRX 2.9.1.1064)	Molecular Devices	https://www.moleculardevices.com/products/cellular-imaging-systems/acquisition-and-analysis-software/cellreporterxpress
MATLAB 2017-2021	Mathworks	https://www.mathworks.com/products/matlab.html
Fiji (ImageJ 1.53f51)	NIH	https://ImageJ.net/software/fiji/
Python 3.6	Python	https://www.python.org/downloads/release/python-360/
Microsoft Excel 2016	Microsoft	https://www.microsoft.com/en-us/microsoft-365/excel
Neuroinfo (2021.1.5)	MBF Biosciences	https://www.mbfbioscience.com/neuroinfo
Graphpad Prism 9	GraphPad	https://www.graphpad.com/scientific-software/prism/
SPSS27	IBM	https://www.ibm.com/analytics/spss-statistics-software
Leica LAS X imaging software	Leica	https://www.leica-microsystems.com/products/microscope-software/p/leica-las-x-ls/
Deposited code	This study	https://zenodo.org/badge/latestdoi/568299290
Deposited code	This study	https://zenodo.org/badge/latestdoi/568299908
Suite2p	https://www.suite2p.org/	SCR_016434
Psychtoolbox	http://psychtoolbox.org	RRID:SCR_002881
Other		
Eagle’s Minimum Essential Medium (EMEM)	ATCC	Cat # 30-2003
Fetal bovine serum	ATCC	Cat # 30-2020

## Highlights

- Excitatory-inhibitory synapse ratio favors neuronal hyperactivity in amyloidosis
- Hyperactivity disrupts functional connectivity and network architecture
- Non-specific visual experience interferes with visual familiarity in amyloidosis
- Non-specific visual experience improves familiarity in non-pathological conditions
