# Supplementary material for: Excitation-inhibition imbalance disrupts visual familiarity in amyloid and non-pathology conditions
Source: Cell Rep. Author manuscript; Available in PMC 2023 Feb 20. (PMC9939293; doi:10.1016/j.celrep.2022.111946)
Supplement: 1 [file NIHMS1870512-supplement-1.pdf]

Cell Reports, Volume *42*

Supplemental information

Excitation-inhibition imbalance disrupts visual familiarity in amyloid and non-pathology conditions

Suraj Niraula, Julia J. Doderer, Shreya Indulkar, Kalen P. Berry, William L. Hauser, Oliver J. L'Esperance, Jasmine Z. Deng, Griffin Keeter, Adam G. Rouse, and Jaichandar Subramanian

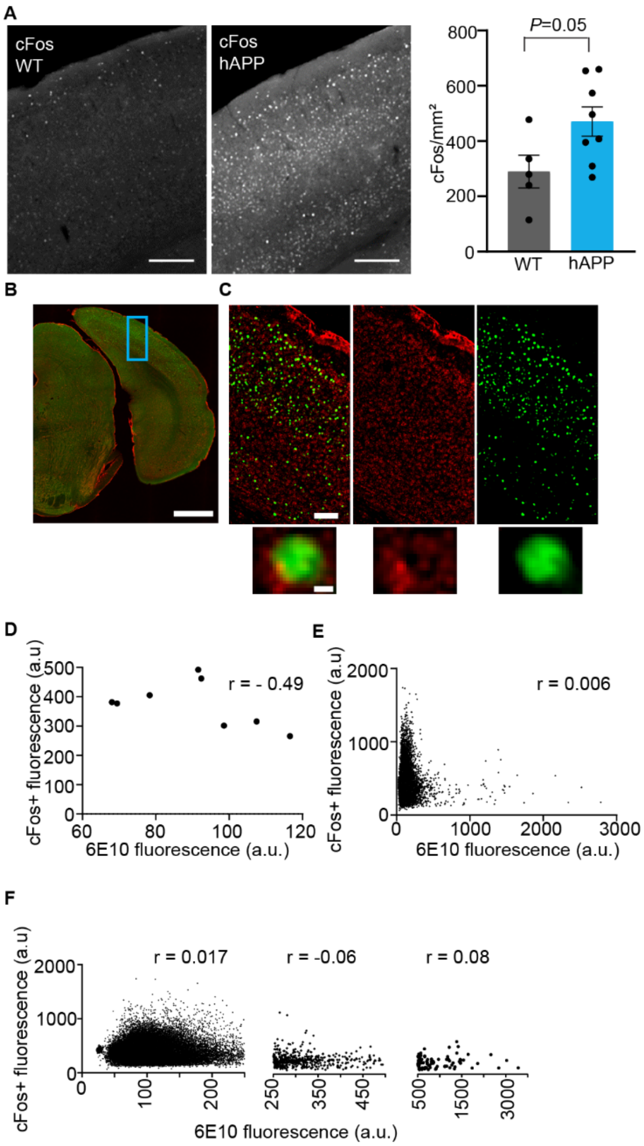

Figure S1. cFos is increased in the visual cortex of hAPP mice. Related to Figure 1.

(**A)** Representative immunohistochemistry for cFos in WT (left) and hAPP (middle) slice. Quantification of cFos density (right). Display ranges are identical for WT and hAPP slices. Scale: 200 µm. Data are mean ± SEM. p = 0.05, unpaired Student's *t*-test. *n* = 5 (WT), 8 (hAPP) mice. Circles in the histogram represent individual mouse values. (**B)** A representative image of a posterior cortical slice from an hAPP mouse stained for cFos and 6e10. Scale: 1 mm. The blue box represents a part of the visual cortex zoomed in C. **(C)** Merged (left), 6e10 (red; middle), and cFos (green; left) immunohistochemistry in hAPP mice (scale: 100 µm). Bottom: a single neuron

zoomed in from the imaged region on top. Scale: 4 µm. **(D)** Correlation of average cFos and 6e10 immunofluorescence (arbitrary units (a.u.) of the visual cortical slices from each mouse. Circles represent individual mouse values (n = 8 hAPP mice). **(E)** Correlation of cFos and 6e10 immunofluorescence of neurons from these mice. Circles represent individual neuron values (58,659 neurons). **(F)** Same as (E) except that the X-axis is separated into different bins.

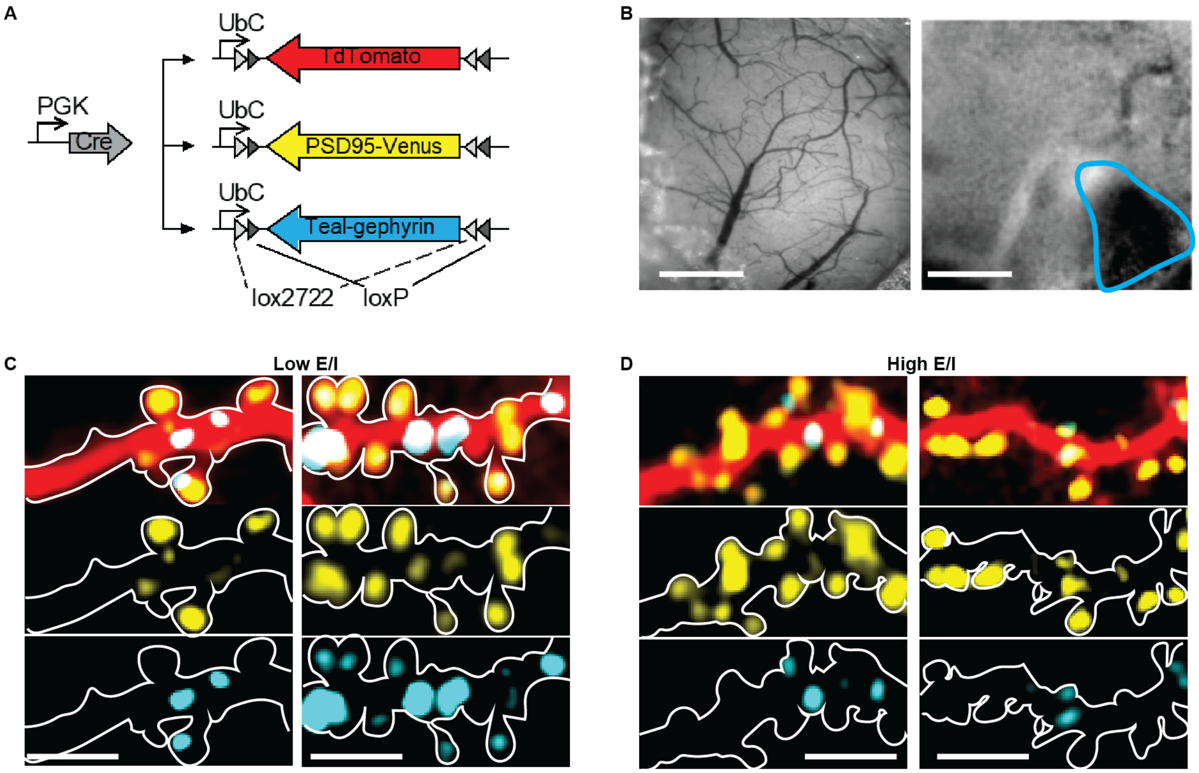

Figure S2. In vivo imaging of E/I ratio. Related to Figure 1.

(A) Cre recombinase-dependent labeling system. PGK, UbC are promoters that drive the expression of the genes listed in the arrows. The open reading frame (ORF) is placed in an inverse orientation between double inverted orientation lox sequences (represented by arrowheads). Cre recombinase inverts the ORF such that it can be expressed from the promoter. (**B)** A representative image of a cranial window with the vasculature (left) and the magnitude map of intrinsic signal (within the blue lines) to identify the location of the visual cortex. Scale: 1 mm. (**C-D)** Two representative images each for low E/I (**C**) and high E/I dendritic segments (**D**). Scale: 5 μm.

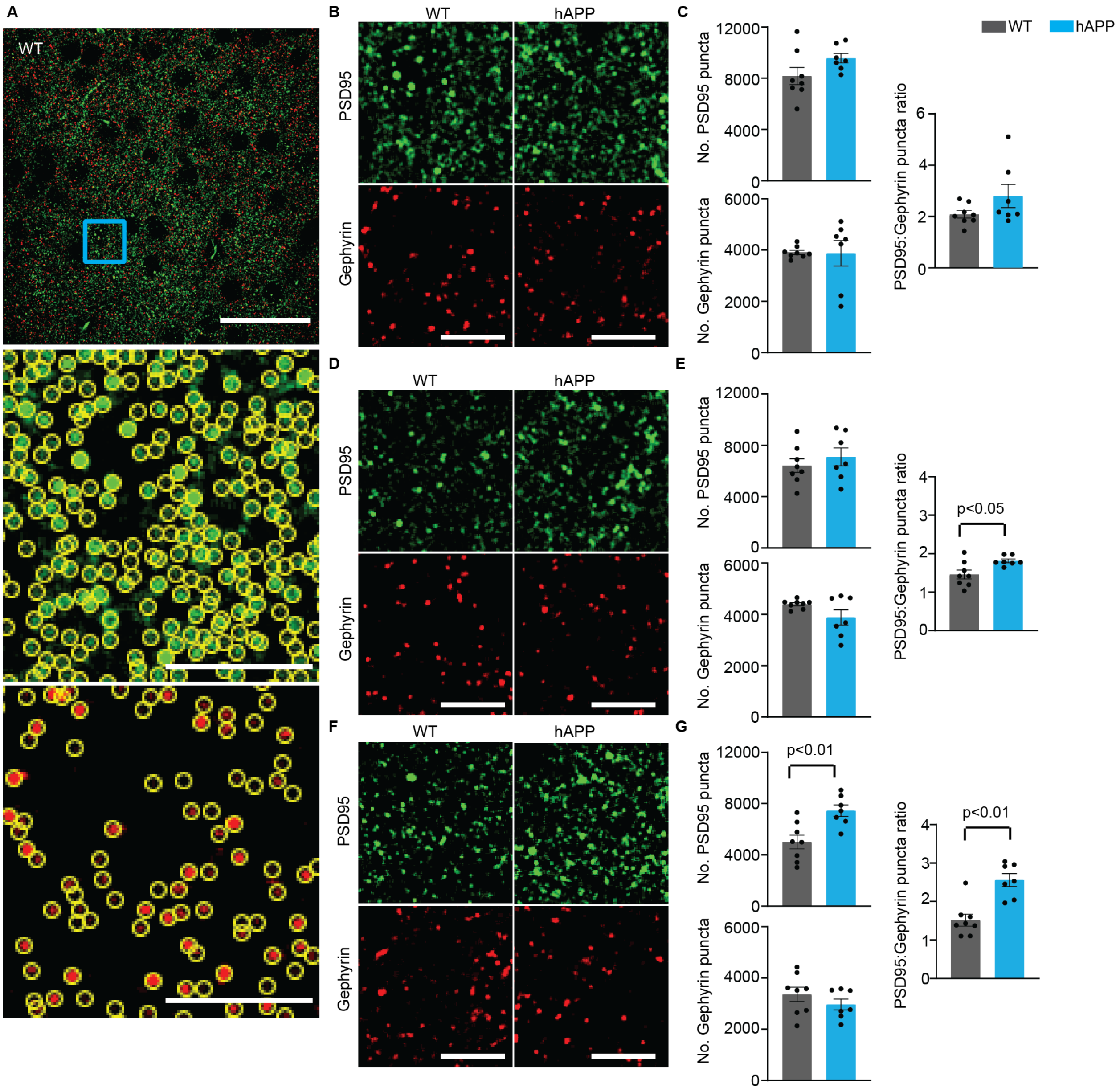

Figure S3. PSD95:Gephyrin ratio is increased in the visual cortex and the hippocampus. Related to Figure 1.

(A) A representative image of 174c x 174µm of superficial visual cortex from wild type (WT) mice with PSD95 (green) and gephyrin (red) puncta. Scale: 50 µm. The blue square is zoomed below to represent puncta detection in PSD95 (middle) and gephyrin (bottom) channels. Scale: 10 µm. The same region is represented in (B) for WT. **(B, D, F)** Representative zoomed in

images of WT and hAPP visual cortex (B), *stratum radiatum* (D), and *stratum oriens* of CA1 (F) immunofluorescence for PSD95 (top) and gephyrin (bottom). Scale: 10 µm. **(C, E, G)** Average number (no.) of PSD 95 (left top), gephyrin puncta (left bottom), and their ratio (right) in 174 µm x 174 µm images from the visual cortex (C), *stratum radiatum* (E), and *stratum oriens* of CA1 (G). $p < 0.05$ (E) and $p < 0.01$ (G), unpaired Student's *t*-tests. $n = 8$ (WT), 7 (hAPP) mice. Circles in the histogram represent individual mouse values.

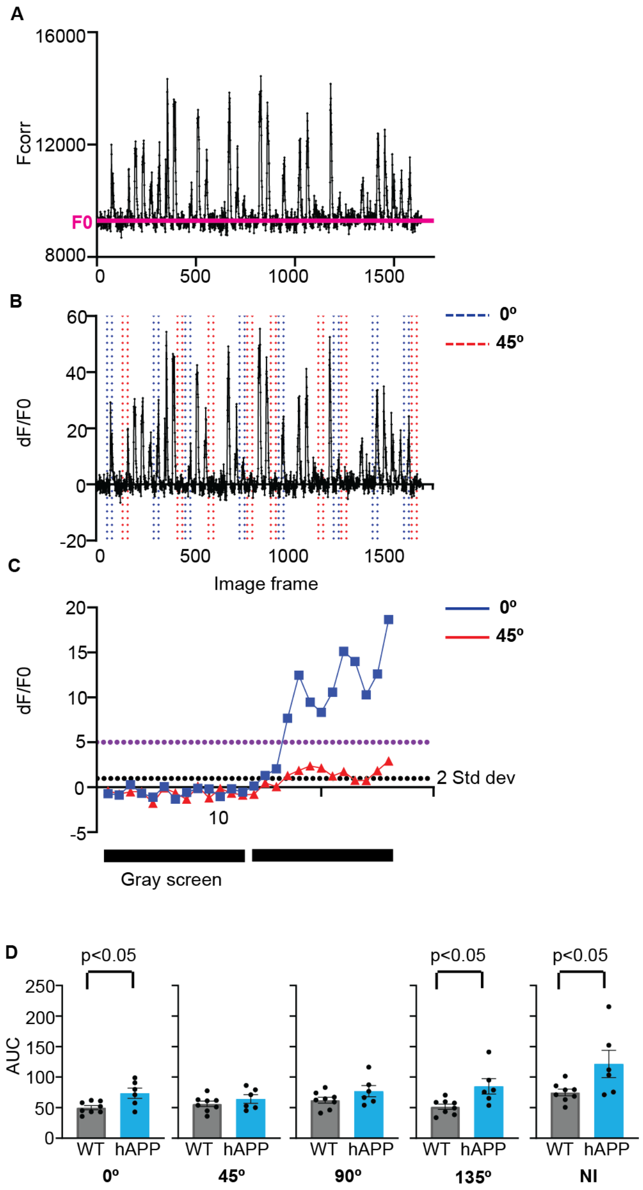

Figure S4. Increased stimulus evoked activity in amyloidosis. Related to Figure 2.

(A) Raw GCaMP6s fluorescence corrected for neuropil contamination (Fcorr) obtained from one cell during ~380 seconds of imaging (1600 frames) with orientation grating stimuli and natural images. The pink line indicates baseline fluorescence (F0). (**B)** dF/F0 trace obtained from **A.** Transients elicited by 0˚ and 45˚ stimuli lie within Blue and red dotted lines, respectively, during eight cycles of stimulus presentation. The timing of other stimuli is not shown. **(C)** Mean dF/F0 obtained from averaging the responses from the eight cycles for each stimulus. Purple (5% dF/F0

– high threshold) and black (2 standard deviations of the gray screen response – low threshold) dotted lines are the thresholds to classify neurons as active. **(D)** Area under the curve (AUC) during three-second stimulus period in neurons (wild type (WT) (479 (0 ˚), 463 (45˚), 529 (90˚), 485 (135˚), and 389 (NI) neurons), 6 (hAPP (182 (0˚), 150 (45˚), 179 (90˚), 160 (135˚), and 155 (NI) neurons) considered active using the low threshold (also includes neurons considered active using high threshold). Data are mean ± SEM. $p < 0.05$, unpaired Student's *t*-tests. $n = 8$ (WT), 6 (hAPP) mice. Circles in the histogram represent individual mouse values.

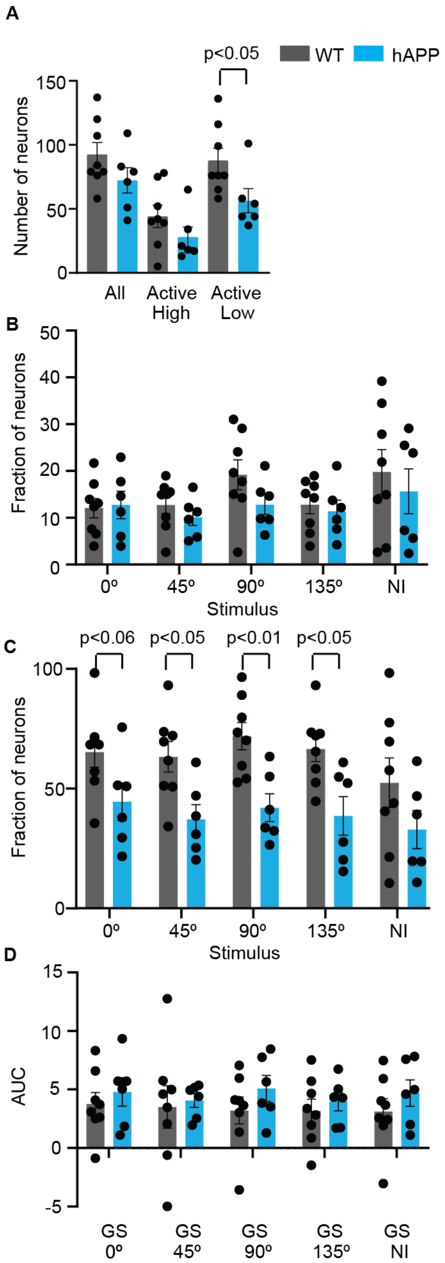

Figure S5. Gray screen response is unaltered, whereas active neurons show a reduction in amyloidosis. Related to Figure 2.

(A) Number of neurons identified as ROIs (all) for analysis did not significantly differ between genotypes. Total visually responsive neurons (active) were lower in hAPP mice but not significantly different when a high threshold (high) was used. However, total visually responsive neurons (number of neurons considered active/all identified neurons) shows a significant reduction in hAPP mice when the threshold is lowered (low) to determine activity. $p < 0.05$,

unpaired Student's *t*-test. (**B-C)** The fraction of neurons (the number of neurons responsive to the given stimulus/all identified neurons) responsive to different visual stimuli (high threshold (B), low threshold (C). Visually responsive neurons are reduced when the threshold for visual responsiveness is lowered. $p < 0.06$ (0˚), $p < 0.05$ (45˚, 135˚), and $p < 0.01$ (90˚), unpaired Student's *t*-tests. (**D)** The area under the curve (AUC) of dF/F0 during the gray screen period preceding any stimuli (listed in the X-axis) is similar in wild type (WT) and hAPP mice. Data are mean ± SEM. $n = 8$ (WT), 6 (hAPP) mice. Circles in the histogram represent individual mouse values.

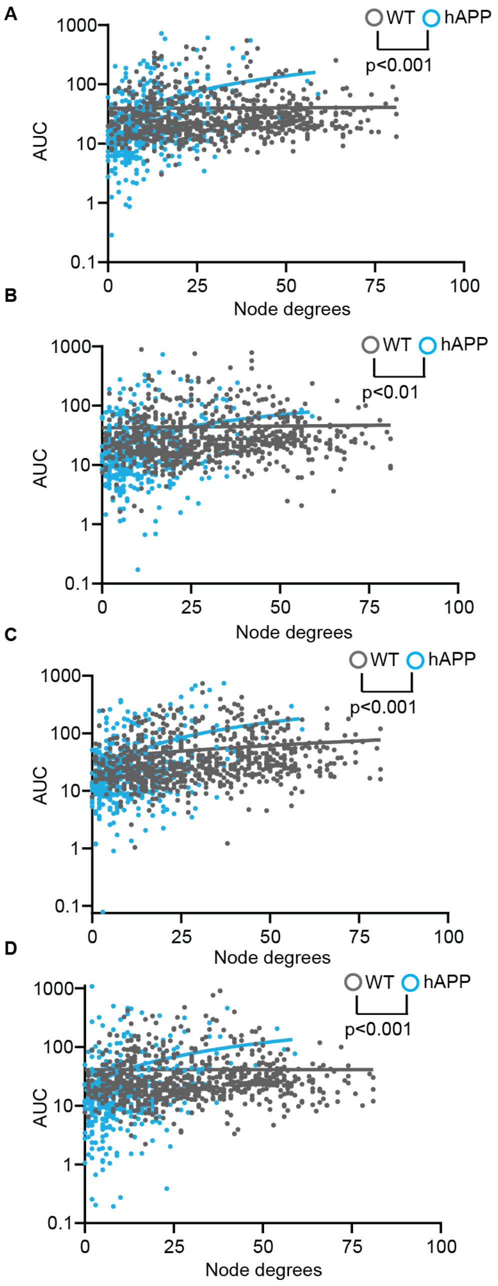

Figure S6. Stimulus responsiveness increases in neurons with higher node degrees in hAPP mice. Related to Figure 4. (A-D) Scatterplot showing the relation between node degrees and the average area under the curve (AUC) during 3 seconds of orientation grating (0 deg (**A**), 45 deg (**B**), 90 deg (**C**), and 135 deg (**D**)) stimuli (Y-axis: log scale). Gray and blue lines (slopes p <

0.001(A, C, D), and $p < 0.01$ (B)) are least-squares fit for wild type (WT) and hAPP mice, respectively.

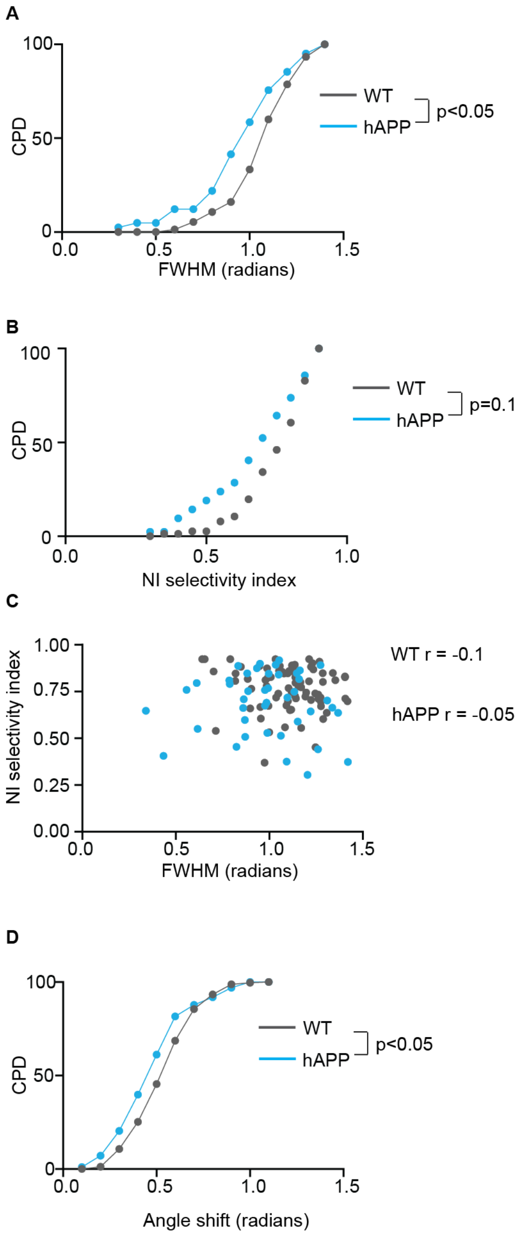

Figure S7. Selectivity of orientation grating and natural image stimuli does not correlate. Related to Figure 4. (A) Cumulative probability distribution (CPD) of full width at half maximum (FWHM) of the tuning curve of neurons that respond to any grating stimuli and natural images (n = 75 (wild type (WT), 41 (hAPP) neurons). $p < 0.05$, Kolmogorov-Smirnov (KS) test. **(B)** Cumulative probability distribution (CPD) of natural image selectivity index of neurons that respond to any grating stimuli and natural images (n = 75 (WT), 41 (hAPP)

neurons). $p = 0.1$, KS test. **(C)** Correlation of FWHM and natural image selectivity index in the same neurons (n = 75 (WT), 41 (hAPP) neurons). Circles represent individual neuron values. No significant correlation was found. **(D)** Cumulative probability distribution (CPD) of difference in preferred orientation of neurons and their functionally connected neurons (n = 242 (WT), 98 (hAPP) neurons). $p < 0.05$, KS test.

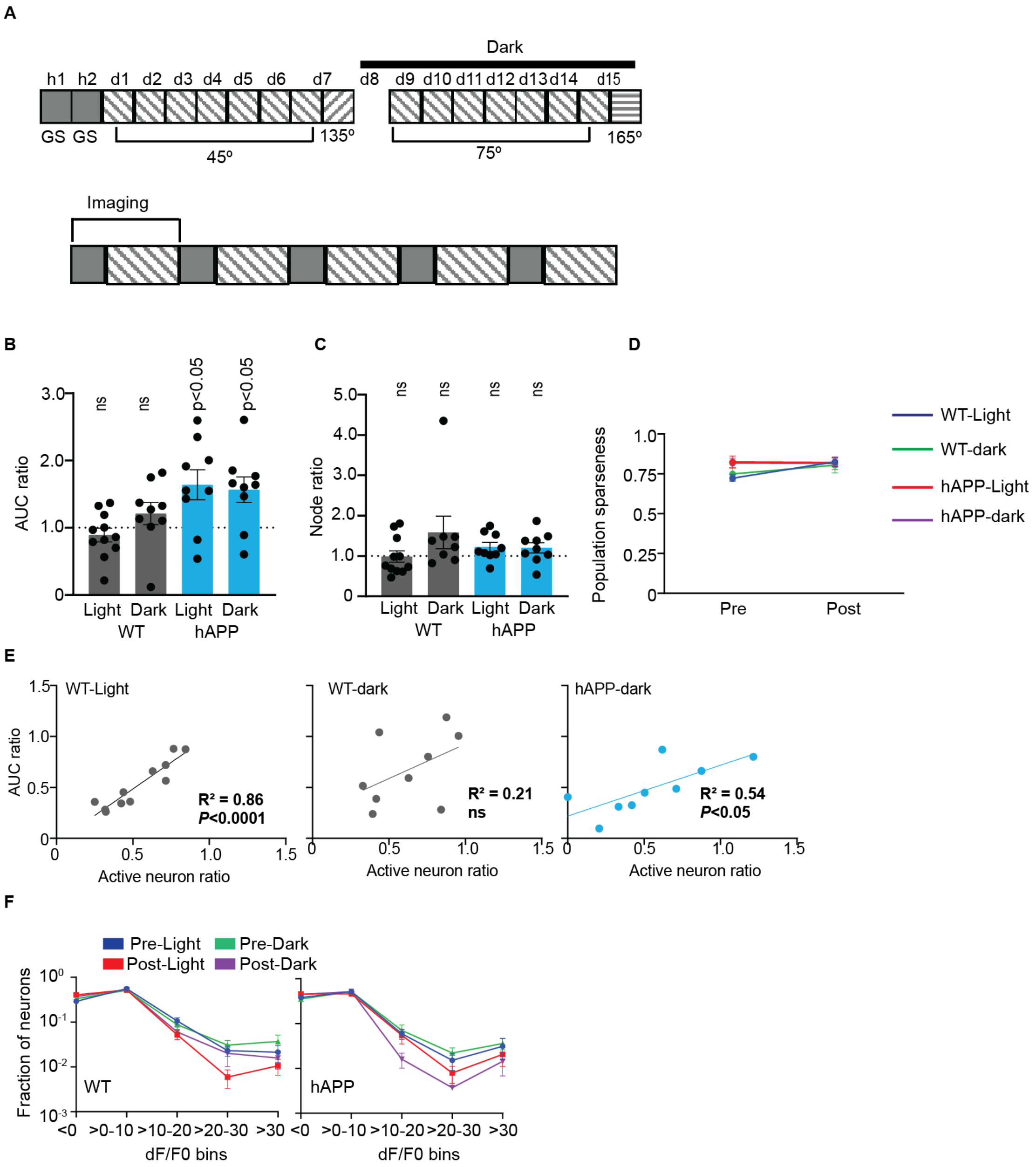

Figure S8. Repeated experience of the same stimulus induces selective plasticity, alters connectivity and plasticity in amyloidosis. Related to Figure 5.

(A) Timeline for the plasticity paradigm. Days (d) and the visual stimuli are indicated. h1,h2 are habituation days. GS – gray screen. The dark bar represents the time for dark housing. On d1, d7, d9, and d15, two photon imaging of neural activity was recorded during the first of the five blocks of gray screen and orientation grating stimulus (bottom). **(B)** The average post to pre training ratio of area under the curve (AUC) during the first ten seconds for novel stimuli. hAPP

mice show increased response to post-novel stimuli ($p < 0.05$, one sample *t*-tests (comparison of the sample mean with the ratio of 1)) because the slightly elevated response to 135˚ (compared to 45º) is consistent with the results from the group used to characterize excitability. **(C)** The average post to pre-training ratio of node degrees (number of coactive neurons to each neuron) during the first ten seconds of stimulus for active neurons for novel stimuli in light and dark. Dotted line indicates identical pre and post-training values. ns – not significant ($p > 0.05$). **(D)** Average population sparseness pre- and post-training. Three-way mixed model ANOVA – $p = 0.056$ for Training x Genotype interaction. **(E)** Scatterplot showing the relation between active neuron ratio and population AUC ratio for wild type (WT-light; $p < 0.0001$ (left)), WT-dark (middle), and hAPP-dark; $p < 0.05$ (right) groups. The line represents the least-squares fit. (**F)** The fraction of neurons (Y-axis: log scale) in different dF/F0 bins for WT (top) and hAPP (bottom) mice. Data are mean ± SEM. $n = 11$ (WT-light), 9 (WT-dark), 9 (hAPP-light and dark) mice. Circles in the histogram represent individual mouse values.

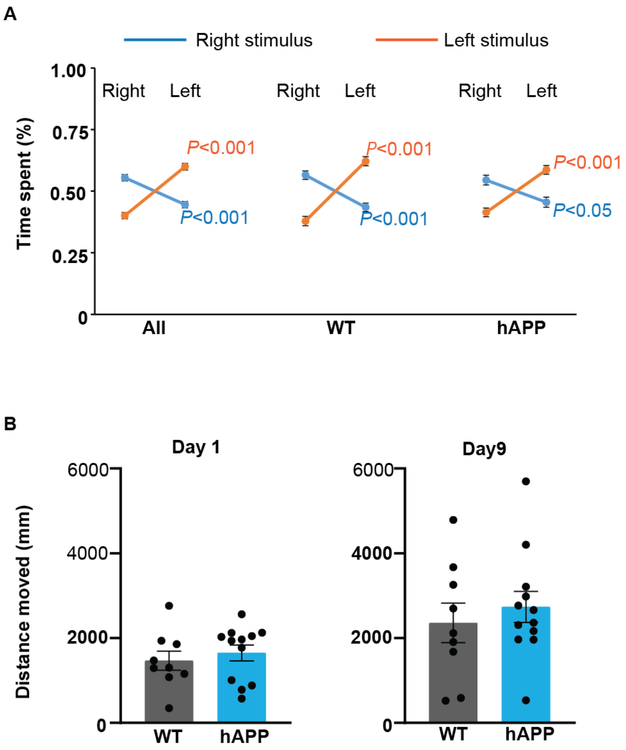

Figure S9. Visual exploration difference in hAPP mice is independent of stimulus location and total movement. Related to Figure 6.

(A) Percentage of time spent exploring the right and left zones when the stimulus was presented on the right (blue line) or left (orange line) monitors for all mice (wild type (WT) and hAPP pooled data; $p < 0.001$ (left and right stimuli)), WT ($p < 0.001$ (left and right stimuli)) and hAPP mice (($p < 0.001$ (left stimulus) and $p < 0.05$ (right stimulus)), paired Student's *t*-tests. **(B)** Distance moved in the chamber in one session. Data are mean ± SEM. Right stimulus $n = 23$ (all); 11 (WT); and 12 (hAPP) sessions. Left stimulus $n = 19$ (all); 7 (WT); and 12 (hAPP) sessions **(A)**. $n = 9$ (WT-light) aand 12 (hAPP-light) mice. Circles in the histogram represent individual mouse values.

A

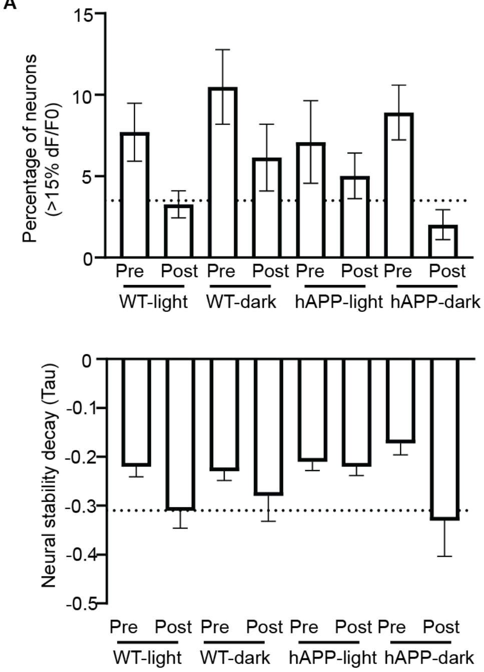

B

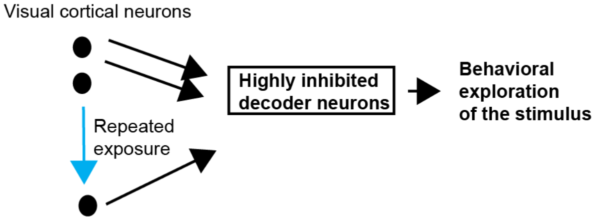

Figure S10. A model for a neural mechanism for visual recognition memory deficit in amyloid and non-pathological conditions. Related to Figure 6.

(A) The average fraction of neurons considered high responding (>15% dF/F0 (corresponding to average dF/F0 of top 20% of neurons for a novel grating stimulus); top) and the average rate of decay of neural representation of visual stimulus (bottom) pre and post-training in WT and hAPP mice housed in light or dark conditions (values from Supplementary Table 2). The conditions that showed more positive values than the dotted line (matched to post-training WT-light) showed increased exploration of visual stimulus, whereas the ones that matched or were more negative than the dotted line showed decreased exploration of the visual stimulus. **(B)** We

propose that the coactivity of a higher fraction of high-responsive neurons and the longer persistence of their activity during the first ten seconds of stimulus experience activates highly inhibited downstream decoder neurons that promote behavioral exploration of the stimulus. Free moving behavioral exploration of the stimulus is interleaved by other behaviors, such as grooming, and therefore, it allows for multiple “first ten seconds” experiences of the stimulus.

When the fraction of high-responsive neurons or the persistence of neural activity goes below the threshold due to repeated experience, decoder neurons will not receive sufficient excitation from the visual cortical neurons, thereby reducing stimulus exploration. In amyloidosis, reduced stimulus specificity to natural visual experiences could lead to continual coactivity of the few highly connected neurons and maintain the fraction of high-responsive neurons and the persistence of neural activity above the threshold for visual familiarity.
